# Bioorthogonal Fluorescence Turn‐On Labeling Based on Bicyclononyne−Tetrazine Cycloaddition Reactions that Form Pyridazine Products

**DOI:** 10.1002/cplu.201900176

**Published:** 2019-05-14

**Authors:** Sebastian J. Siegl, Juraj Galeta, Rastislav Dzijak, Martin Dračínský, Milan Vrabel

**Affiliations:** ^1^ Institute of Organic Chemistry and Biochemistry of the Czech Academy of Sciences Flemingovo nám. 2 166 10 Prague Czech Republic

**Keywords:** cyclic alkynes, cycloaddition reactions, Diels-Alder reactions, fluorogenic probes, tetrazines

## Abstract

Fluorogenic bioorthogonal reactions enable visualization of biomolecules with excellent signal‐to‐noise ratio. A bicyclononyne−tetrazine ligation that produces fluorescent pyridazine products has been developed. In stark contrast to previous approaches, the formation of the dye is an inherent result of the chemical reaction and no additional fluorophores are needed in the reagents. The crucial structural elements that determine dye formation are electron‐donating groups present in the starting tetrazine unit. The newly formed pyridazine fluorophores show interesting photophysical properties the fluorescence intensity increase in the reaction can reach an excellent 900‐fold. Model imaging experiments demonstrate the application potential of this new fluorogenic bioorthogonal reaction.

Visualization of biomacromolecules involved in biological processes is an important part of biological research. The discovery and development of fluorescent proteins has revolutionized our ability to illuminate subcellular organization of enzymes and proteins and provided us with the unique possibility to study their structure and function in living systems.^1^ Besides these genetically‐encodable methods, the use of small‐molecule fluorescent dyes has further advanced our understanding of biology and extended our possibility to examine biomolecules beyond proteins and enzymes.^2^ Among the main advantages of using small‐molecule fluorophores for bioimaging applications belong their superior and tunable photophysical properties and their small size.

A good signal‐to‐noise ratio is an important metric for determining the success of bioimaging experiments. Accordingly, the development of fluorogenic probes enabling turning‐on of the fluorescence of the reporter molecule in response to a specific molecular event represents significant achievement in suppressing the undesired background signal.^3^ In the last decades, the concept of fluorogenicity was successfully applied to various bioorthogonal reactions, which led to the development of chemically‐activatable fluorogenic probes. These probes usually comprise specific structural features that lead to quenching of the fluorescence of the attached fluorophore. The fluorescence of the dye is then restored after the chemical reaction. This powerful concept was successfully utilized for numerous dyes and bioorthogonal reactions.^4^

Among other bioconjugations, the inverse electron‐demand Diels‐Alder reaction (iEDDA) of 1,2,4,5‐tetrazines with strained dienophiles stands out due to expedient kinetics and excellent biocompatibility.^5^ The inherent properties of the heterocyclic tetrazine core enabled development of fluorogenic tetrazine probes with tunable photophysical properties for bioimaging application.^6^ In addition, we and others have shown that the iEDDA reaction of tetrazines with particular dienophiles can directly lead to formation of fluorescent dyes.^7^ The latter approach represents an attractive alternative to the fluorophore‐quenching concept as the only reaction products are the fluorescent species formed during the reaction and no additional fluorophores are required in the structure of reagents.

In our recent work we showed that 1,2,4,5‐tetrazines decorated with vinylaniline or azetidine electron‐donating groups produce in reaction with various *trans*‐cyclooctenes (TCOs) fluorescent 4,5‐dihydropyridazines.^8^ We found that this class of tetrazines also forms fluorescent products in reaction with the bicyclononyne (BCN) dienophile. This unexpected observation prompted us to study this fluorogenic reaction in more detail.

Here we show that BCN yields fluorescent pyridazines in the reaction with 1,2,4,5‐tetrazines bearing electron‐donating groups (Figure 1). The increase in fluorescence intensity after the reaction can reach up to 900‐fold, which is among the highest values reported for a bioorthogonal reaction to date. This new BCN−tetrazine reaction can be used in combination with the TCO−tetrazine cycloaddition for two‐color fluorogenic imaging. In addition, the fluorogenicity is preserved in biological systems and enables the reaction to be used for bioimaging application.

**Figure 1:**
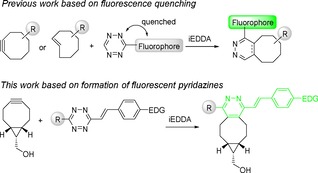
General concept of fluorogenic inverse electron‐demand Diels‐Alder reactions using strained dienophiles and 1,2,4,5‐tetrazines. EDG=electron‐donating group.

We started our study with the reaction of the *p*‐vinyldimethylaniline substituted tetrazine **1 a** with BCN. The HPLC‐MS analysis of the crude reaction mixture showed formation of the signal having the mass corresponding to the expected pyridazine **2 a** (Figure S1 in the Supporting Information). The structure of the product was confirmed by NMR analysis (see the Supporting Information). The absorption maximum of the product is at 376 nm and emission at 506 nm, respectively, giving a 130 nm Stokes shift in CH_3_CN (Figure 3A). The increase in the fluorescence signal after the reaction was 100‐fold (starting tetrazine vs. the click product).

**Figure 2:**
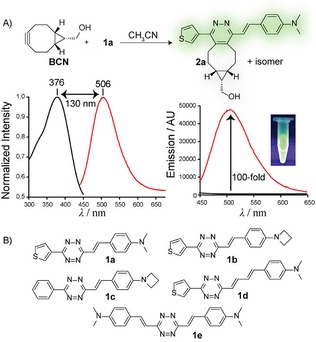
A) An example of the experiment showing formation of the fluorescent pyridazine product in the reaction of BCN with tetrazine **1 a**. Shown are normalized absorption (black) and emission (red) spectra and the fluorescence enhancement after the reaction. Only one isomer (enantiomer) of the product is shown for clarity. B) Structures of tetrazines used in this study.

**Figure 3:**
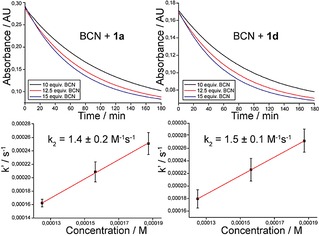
Second‐order rate constant measurements of the reaction between tetrazines **1 a** and **1 d** with BCN. The reactions were performed in 1/1 mixture of CH_3_CN and H_2_O at room temperature (22°C) under pseudo‐first‐order conditions using an excess of BCN.

To study the effect of various substituents on the photophysical properties of the click products we next performed similar experiments with different tetrazines (Figure 2B, Table 1, Table S2). We did not find significant difference in the absorption and emission maxima when using tetrazine **1 b**, which contains azetidine instead of the dimethylamino group. However, the enhancement of the fluorescent signal after the reaction increased to 185‐fold. Tetrazine **1 c** containing the phenyl substituent instead of the thiophene showed in the reaction a further increase in the fluorescence intensity which was 330‐fold. In an attempt to shift the absorption and emission maxima by extending the conjugated π‐system of the fluorophore we next introduced another double bond between the tetrazine core and the substituent bearing the electron‐donating dimethylamino group. Indeed, the absorption and emission maxima of the click product formed in reaction of **1 d** with BCN were 392 nm and 562 nm, respectively.

**Table 1:** Photophysical properties of the click products **2 a**–**2 e** formed in reaction of tetrazines **1 a**–**1 e** with BCN in CH_3_CN.

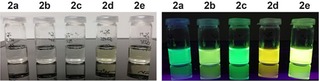				
Click product	*λ*_Abs_/*λ*_Em_ [nm]^[a]^	Stokes shift [nm]	*φ* ^[b]^	Fl. intensity increase
**2 a**	376/506	130	0.014	100‐fold
**2 b**	370/513	143	0.010	185‐fold
**2 c**	369/508	139	0.011	330‐fold
**2 d**	392/562	170	0.038	10‐fold
**2 e**	397/529	132	0.134	900‐fold

[a] Absorption and emission maxima in CH_3_CN. [b] Fluorescence quantum yields were determined using quinine sulfate in 0.5 M H_2_SO_4_ as standard (*φ*=0.55). For copies of absorption and emission spectra see Figure S3–S7.

**Figure 4:**
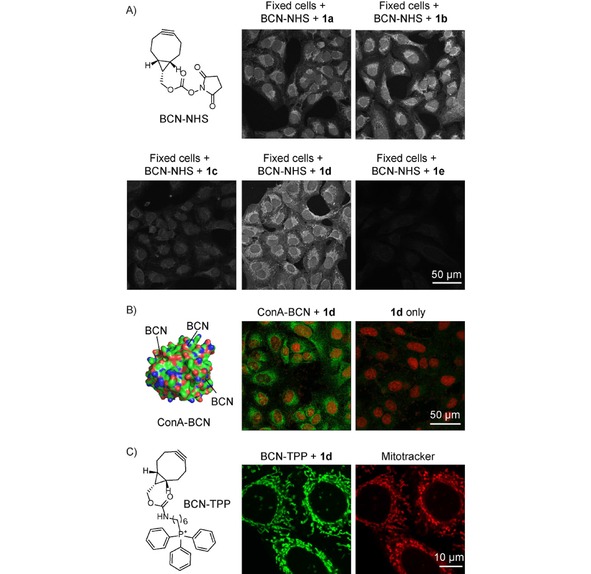
Images from confocal fluorescence microscope showing A) fluorogenic labeling of BCN‐modified HeLa cells after addition of tetrazines **1 a**–**1 e**. Click product excitation: *λ*=405 nm, emission: *λ*=517–587 nm for **1 a**, **1 b**, **1 c** and **1 e** and *λ*=543–639 nm window for **1 d**. B) ConA−BCN modified U2OS cells after addition of **1 d**. Nucleus was stained using commercially available DRAQ5 dye (Excitation: *λ*=633 nm, Emission: *λ*=643–703 nm window). C) fluorogenic labeling of live HeLa cells using BCN−TPP and **1 d**. Mitotracker: Excitation: *λ*=633 nm, Emission: *λ*=640–735 nm window. The pictures are in pseudocolors.

The above experimental data led us to hypothesize that the pyridazine core of the click product serves as an electron‐poor part of the molecule. The presence of electron‐donating substituents then leads to the formation of a push‐pull‐like system, which finally results in formation of the fluorophore. We therefore speculated that introducing the vinylaniline substituent from both sides of the tetrazine core could improve the photophysical properties of the click product. To test this hypothesis we prepared symmetric tetrazine **1 e** and measured the photophysical properties of the click product formed after the reaction with BCN.

Indeed, we found that **1 e** yields a fluorescent product displaying interesting photophysical properties. The absorption and emission maxima of **2 e** were 397 and 529 nm, respectively. Most importantly, the fluorescence enhancement in the reaction of this particular tetrazine with BCN reached an impressive 900‐fold, which is the highest in the series. The fluorescence quantum yield of **2 e** is also the highest among the click products (Table 1).

To simulate more biologically‐relevant conditions we also measured the photophysical properties of the click products **2 a–2 e** in 1/1 mixture of CH_3_CN and H_2_O (Table S3 and Figure S8–S12). Interestingly, tetrazines **1 a**, **1 b** and **1 d** showed even higher fluorescence turn‐on in this solvent mixture, while for **1 c** and **1 e** the fluorescence intensity decreased. However, **2 e** showed higher fluorescence in 1/1 mixture of CH_3_CN and PBS buffer (Figure S12). We also noticed that the absorption and emission maxima of the click products were shifted in CH_3_CN/H_2_O mixture indicating that the pyridazine fluorophores are solvatochromic. We therefore determined the photophysical properties of the click product **2 a** in different solvents. The experiment confirmed this assumption and the absorption and especially the emission maximum as well as the fluorescence quantum yields varied in different solvents (Table S4 and Figure S13–S17). These data indicate that the pyridazines could serve as useful fluorescent environment‐sensitive probes.^9^ In combination with the excellent fluorescence turn‐on properties, these probes could find utility in various imaging applications, where a good signal‐to‐noise ratio is desirable.

The reactivity of 1,2,4,5‐tetrazines with the BCN dienophile usually differs from that with the TCOs.5c,^10^ To gain insight into the reactivity of our group of tetrazines with BCN we measured the second order rate constant for two representative derivatives, namely for **1 a** and **1 d**. The second order rate constants were determined at room temperature (22°C) in a 1/1 mixture of CH_3_CN and H_2_O under pseudo first‐order conditions using an excess of BCN by following the decay in the absorption of the starting tetrazine over time (Figure 3). The observed rate constants for each BCN concentration were finally plotted against BCN concentration and the second order rate constants were calculated from the slope of the plot (Table S5). The experiments were performed in triplicate. The determined second order rate constant for the reaction of tetrazine **1 a** with BCN was 1.4±0.2 M^−1^ s^−1^ and 1.5±0.1 M^−1^ s^−1^ for tetrazine **1 d**, respectively. These data show that there is no significant difference in reactivity of these two tetrazines.

For comparison, similar tetrazines react about ten‐times faster with the equatorial *trans*‐cyclooctenol as we determined in our previous work.^8^ On the other hand, the determined reaction rate of the BCN−tetrazine cycloaddition is comparable to that of the reaction of tetrazines with, for example, norbornenes,^11^ which are useful bioconjugation reagents and have been successfully used in bioimaging applications.^12^

We next studied the fluorogenic reaction of tetrazines **1 a**–**1 e** with BCN on HeLa cells. For this purpose, the cells were fixed and treated with the BCN active ester. We then added the individual tetrazines and inspected the cells on a confocal fluorescence microscope (Figure 4A and Figure S18). Addition of tetrazine **1 e**, which we found superior regarding its photophysical properties (Table 1), led to formation of only a very weak fluorescent signal in the cells. Similarly **1 c** also did not form significant labeling pattern. Tetrazines **1 a** and **1 b** performed similarly and gave rise to a visible fluorescent signal. Among the tetrazines tested, compound **1 d** was found superior and we observed formation of clear visible fluorescent signal in the cells. Importantly, we did not observe any fluorescent signal in cells treated only with the tetrazines showing that formation of the fluorescent signal is a result of the reaction (Figure S18).

To further probe the fluorogenic reaction on cells we prepared concanavalin A modified with the BCN moiety (ConA−BCN). ConA is a lectin extensively used in studies of glycoconjugates.^13^ After incubation of U2OS cells with ConA−BCN and addition of tetrazine **1 d** we observed formation of a fluorescent signal indicating successful reaction, while cells treated only with the tetrazine were not fluorescent (Figure 4B).

To examine the fluorescence turn‐on reaction in live cells we prepared BCN−triphenylphosphonium conjugate (BCN−TPP). The TPP moiety was used in order to target the BCN group to mitochondria.^14^ Live HeLa cells were incubated with BCN−TPP and the fluorogenic reaction was subsequently initiated by addition of **1 d**. Analysis of the cells by confocal microscopy showed formation of fluorescent signal inside mitochondria as confirmed by co‐staining with commercially available mitotracker deep red dye (Figure 4C). Importantly, cells treated only with **1 d** were not fluorescent. We obtained similar results with live U2OS cells (Figure S18).

We also evaluated the toxicity of **1 a**–**1 e** on HeLa and U2OS cells using XTT or crystal violet assay. Our results show that the tetrazines are not toxic up to 50 μM concentration after 24 hours of incubation (Figure S20).

Our recent discovery that various TCOs form 4,5‐dihydropyridazine fluorophores in reaction with the same group of tetrazines^8^ led us to speculate that we could use the BCN and the TCO moiety for two‐color fluorogenic labeling using a single tetrazine as the activator. To test this on a model system, we prepared a topologically segregated bilayer Tentagel (TG) resin beads.^15^ The outer layer of the beads was modified with the BCN moiety, while the inner part was functionalized by the TCO.

As the two fluorophores build from the two dienophiles (fully aromatic pyridazine from BCN and 4,5‐dihydropyridazine from TCO) have distinct photophysical properties they should be distinguishable by using different excitation wavelength and emission filters.^8^ Indeed, when we incubated the BCN−TCO‐modified TG beads with tetrazine **1 a** we observed clear formation of two fluorophores confirming a successful reaction and fluorescence turn‐on corresponding to the reaction of the tetrazine with the two different dienophiles (Figure 5 and Figure S19). This model experiment demonstrates that the inherent fluorogenic nature of the TCO− and BCN−tetrazine click reaction can be utilized and combined on one system enabling two‐color fluorogenic labeling after addition of a single tetrazine. However, the tautomerization of the 4,5‐dihydropyridazine to the corresponding 1,4‐dihydropyridazine as well as the propensity of both isomers to form oxidized pyridazines must be carefully considered in similar experiments as this may lead to changes in the fluorescence over time (Figure S21).^8^,^16^

**Figure 5:**
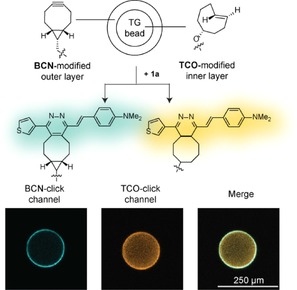
Confocal fluorescence microscope images showing two‐color fluorogenic labeling of bilayer segregated Tentagel beads containing the BCN moiety on the outer layer and the TCO moiety in the inner part of the beads. BCN‐click channel: Excitation at *λ*=458 nm, Emission at *λ*=508–556 nm window. TCO‐click channel: Excitation at *λ*=561 nm, Emission at *λ*=575–645 nm window. The pictures are in pseudocolors.

In this study, we have shown that tetrazines decorated with electron‐donating substituents connected via π‐system yield in reaction with the strained bicyclononyne dienophile fluorescent pyridazine dyes. The fluorophores formed have large Stokes shift, but rather low fluorescence quantum yield. The fluorescence turn‐on in the reaction exceeded 100‐fold in most cases and reached an impressive 900‐fold when symmetric double‐substituted tetrazine was used. The fluorogenic nature of the reaction is preserved in biological systems and provides application to bioimaging. In combination with the TCO−tetrazine cycloaddition, the reaction enables simultaneous two‐color fluorogenic labeling using a single tetrazine as the activator.

## Conflict of interest

The authors declare no conflict of interest.

## Supporting information

As a service to our authors and readers, this journal provides supporting information supplied by the authors. Such materials are peer reviewed and may be re‐organized for online delivery, but are not copy‐edited or typeset. Technical support issues arising from supporting information (other than missing files) should be addressed to the authors.

Supplementary
