# Supplementary material for: Bioorthogonal Fluorescence Turn‐On Labeling Based on Bicyclononyne−Tetrazine Cycloaddition Reactions that Form Pyridazine Products
Source: Chempluschem. 2019 May 14;84(5):493–7. doi: 10.1002/cplu.201900176 (PMC6582594; doi:10.1002/cplu.201900176)

CHEMPLUSCHEM

Supporting Information

© Copyright Wiley-VCH Verlag GmbH & Co. KGaA, 69451 Weinheim, 2019

Bioorthogonal Fluorescence Turn-On Labeling Based on Bicyclononyne–Tetrazine Cycloaddition Reactions that Form Pyridazine Products

Sebastian J. Siegl, Juraj Galeta, Rastislav Dzijak, Martin Dračínský, and Milan Vrabel*© 2019 The Authors. Published by Wiley-VCH Verlag GmbH & Co. KGaA. This is an open access article under the terms of the Creative Commons Attribution License, which permits use, distribution and reproduction in any medium, provided the original work is properly cited.This article is part of the “Early Career Series”. To view the complete series, visit: .

Content

General information 2
Synthetic procedures........ 3
Isolation and confirmation of click product 2a 4
Photophysical properties of the click products of BCN with tetrazines 1a-1e........ 5
Determination of second-order rate constants 18
Fluorogenic cell labeling 19
Two-color fluorogenic labeling of segregated bilayer TG beads........ 22
Toxicity studies 23
Time-lapse labeling of BCN- and TCO-modified TG beads 25
References........ 27
Copies of NMR spectra 28

General information

The chemicals were obtained from commercial suppliers and were used without further purification. Reactions with air- and moisture-sensitive reactants were performed in anhydrous solvents under nitrogen or argon atmosphere. Column chromatography was carried out on silica gel 60A (particle size: 40-60 µm) from *Acros Organics*. Mixtures of solvents are each stated as volume fractions. For flash column chromatography a Combi*Flash*® Rf+ from *Teledyne ISCO* was used. Thin-layer chromatography was performed on aluminum sheets from *Merck* (silica gel 60 F254, 20 × 20 cm). Chromatograms were visualized by UV light (λ = 254 nm/ 366 nm) or by staining with $KMnO_4$ solution. ^{1}H- and ^{13}C-NMR spectra were measured on a Bruker Avance III™ HD 400 MHz NMR system equipped with Prodigy cryo-probe. Chemical shifts δ are quoted in ppm in relation to the chemical shift of the residual non-deuterated solvent peak ($CDCl_3$: δ(^{1}H) = 7.26, δ(^{13}C) = 77.16; DMSO-d_6: δ(^{1}H) = 2.50, δ(^{13}C) = 39.52). High-resolution mass spectra were recorded on an *Agilent* 5975C MSD Quadrupol, Q-Tof micro from *Waters* or LTQ Orbitrap XL from *Thermo Fisher Scientific*. HPLC-MS measurements were performed on an LCMS-2020 system from *Shimadzu* equipped with a Luna® C18(2) column (3µm, 100A, 100 × 4.6 mm). UV/VIS spectroscopy was performed on a Cary 60 UV/Vis spectrophotometer from *Agilent Technologies*. Data from kinetic experiments were processed using OriginPro 9.1 software. Fluorescence measurements were performed on a FluoroMax 4 spectrofluorometer (Jobin Yvon, Horiba) from *Perkin Elmer* equipped with a 450 W xenon lamp and a single cuvette reader using the dye quinine sulfate (solution in 0.5 M H_2SO_4) as standard for determination of fluorescence quantum yields.

Synthetic procedures

The synthesis of tetrazines **1a**[1] and **1b**,**c**[2] has already been published in our earlier publications.

Synthesis of tetrazines 1d, 1e and BCN-TPP

N,*N*-Dimethyl-4-((1*E*,3*E*)-4-(6-(thiophen-3-yl)-1,2,4,5-tetrazin-3-yl)buta-1,3-dien-1-yl)aniline (1d)

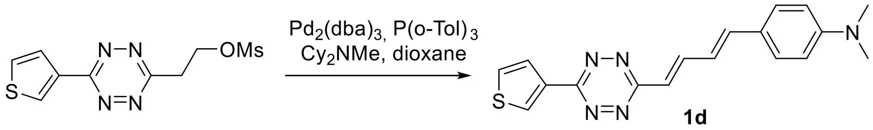

To a Schlenk vessel was added mesylated starting tetrazine[1] (100 mg, 0.349 mmol), P(*o*-Tol)$_3$ (43 mg, 0.140 mmol, 40 mol%), $Pd_2(dba)_3$ (32 mg, 0.0349 mmol, 10 mol%) and (*E*)-4-(2-iodovinyl)-*N*,*N*-dimethylaniline[3] (224 µL, 1.05 mmol, 1.5 equiv). The vessel was purged with argon and 10 mL of degassed anhydrous 1,4-dioxane was added via syringe followed by Cy_2NMe (3 equiv). The reaction mixture was vigorously stirred at 90 °C and cooled to RT after 20 h. Water (10 mL) was added and the mixture was extracted with DCM (3 × 5 mL), the combined extracts were dried over Na_2SO_4, filtered and evaporated. Column chromatography (1. DCM; 2. DCM/PE 3:1) and subsequent sonication of the residue with acetone and decanting afforded 48 mg (41%) of a dark red tetrazine **1d**.
^{1}H NMR (400 MHz, $CDCl_3$): 8.54 (dd, 1H, *J* = 3.0, 1.2), 8.05–8.12 (m, 1H), 8.04 (dd, 1H, *J* = 5.1, 1.2), 7.50 (dd, 1H, *J* = 5.1, 3.0), 7.43 (d, 2H, *J* = 8.9), 6.93–6.96 (m, 2H), 6.90 (d, 1H, *J* = 15.4), 6.69 (d, 2H, *J* = 8.9), 3.02 (s, 6H).
^{13}C NMR (101 MHz, $CDCl_3$): 164.9, 160.2, 151.2, 142.6, 141.4, 135.5, 129.3, 129.0, 127.4, 126.6, 124.4, 123.3, 121.1, 112.2, 40.4.
HRMS (ESI): m/z calcd. for $C_{18}H_{18}N_5S$ $[MH]^+$ 336.1277, found 336.1278.

4,4'-((1*E*,1'*E*)-(1,2,4,5-Tetrazine-3,6-diyl)bis(ethene-2,1-diyl))bis(*N*,*N*-dimethylaniline) (1e)

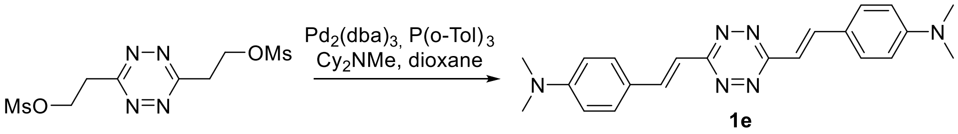

To a Schlenk vessel was added bis-mesylated tetrazine[4] (100 mg, 0.306 mmol), P(*o*-Tol)$_3$ (37 mg, 0.123 mmol, 40 mol%), $Pd_2(dba)_3$ (28 mg, 0.0306 mmol, 10 mol%) and 4-bromo-*N*,*N*-dimethylaniline (184 mg, 0.919 mmol, 3 equiv). The vessel was purged with argon and 10 mL of degassed anhydrous 1,4-dioxane was added via syringe followed by Cy_2NMe (3 equiv). The reaction mixture was vigorously stirred at 90 °C and cooled to RT after 20 h. Water (10 mL) was added and the mixture was extracted with DCM (3 × 5 mL), the combined extracts were dried over Na_2SO_4, filtered and evaporated. Column chromatography (DCM → DCM/EA 10:1) and subsequent sonication of the residue with acetone and decanting afforded 33 mg (29%) of a very dark violet tetrazine **1e**.
HRMS (ESI): m/z calcd. for $C_{22}H_{25}N_6$ $[MH]^+$ 373.2141, found 373.2139.

Unfortunately the product showed poor solubility even in DMSO so that we were unable to get sufficiently concentrated solution to characterize it by NMR. To therefore confirmed its identity indirectly using HPLC/MS by reaction with the BCN dienophile. For this purpose, a suspension of tetrazine **1e** in DMSO was mixed with a solution of BCN in CH_3CN at room temperature using an excess of BCN. The mixture was stirred in the dark for 19 h and then measured by HPLC-MS. HRMS (ESI): m/z calcd. for $C_{32}H_{39}N_4O$ $[MH]^+$ 495.3124, found 495.3128. For details and results of the measurement see Table S1 and Figure S2.

(6-(((bicyclo[6.1.0]non-4-yn-9-ylmethoxy)carbonyl)amino)hexyl)triphenylphosphonium formate (BCN-TPP)

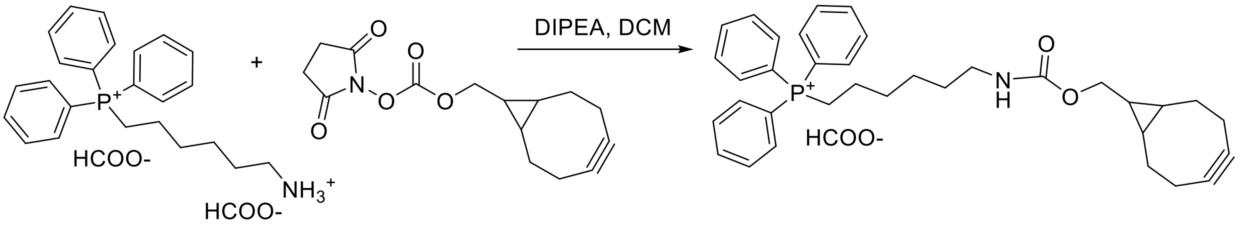

To a solution of BCN active ester (32 mg, 0.11 mmol) and TPP-amine (50 mg, 0.11 mmol, prepared as described in Dyes and Pigments, 2017, 407) in DCM (3 mL) was at 0°C dropwise added DIPEA (96 µL, 5 equiv.) The reaction mixture was stirred for 30-45 min at room temperature when HPLC/MS analysis showed that the reaction finished. After evaporation under reduced pressure, the crude reaction was purified by C-18 RP silica gel flash chromatography using a gradient of CH_3CN in H_2O as the eluent (5% to 80% over 10 min). Fractions containing the product were collected and the solvent was removed under reduced pressure giving the product as white foam (19 mg, 32%).

^{1}H NMR (400 MHz, Chloroform-*d*) δ 7.89 – 7.73 (m, 9H), 7.69 (ddd, *J* = 8.6, 6.9, 3.4 Hz, 6H), 4.08 (m, 2H), 3.81 (m, 2H), 3.12 (q, *J* = 6.4 Hz, 2H), 2.31 – 2.12 (m, 6H), 1.74 – 1.16 (m, 12H), 0.89 (ddd, *J* = 11.0, 6.1, 2.4 Hz, 2H).

^{13}C NMR (101 MHz, $CDCl_3$) δ 157.07, 135.09, 135.06, 133.85, 133.75, 130.64, 130.51, 118.92, 118.07, 98.98, 77.48, 77.36, 77.16, 76.84, 62.45, 53.56, 40.63, 30.00, 29.84, 29.58, 29.19, 26.17, 22.99, 22.63, 22.49, 21.55, 20.19, 17.94.

(ESI): m/z calcd. for $C_{35}H_{41}NO_2P$ $[M]^+$ 538.2869, found 538.2877.

Isolation and confirmation of click product 2a

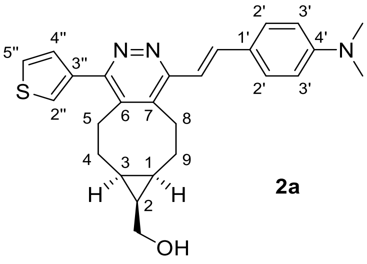

2a

To a solution of tetrazine **1a** (30.0 mg, 0.0970 mmol) in CH_3CN (3 mL) was added bicyclononyne (29.1 mg, 0.194 mmol). The reaction mixture was stirred for 18 h at room temperature until the starting materials disappeared (verified by TLC in DCM/EtOAc 1:1 or HPLC-MS, see Figure S1). The crude product was concentrated *in vacuo* and purified by flash column chromatography (0→40% EtOAc in DCM) to obtain an isomeric mixture of click product **2a** (44 mg). Separation by preparative TLC (eluting with DCM/EtOAc 4:1 + 5% MeOH and elution of the major product from the silica with DCM/MeOH 4:1) provided the main isomer of **2a** as yellow solid (27 mg, 64%).

As the occurrence of rotamers complicated the analysis of the cyclooctene moiety of **2a** at room temperature, the NMR measurements were performed in DMSO-d_6 at 80 °C.

^{1}H NMR (600.1 MHz, DMSO-d_6): δ 7.79 (d, 1H, $C^{1'}$-**CH**=CH, $J_{H\text{-}C=C\text{-}H}$ = 15.6), 7.70 (dd, 1H, $C^{2''}$**H**, $J_{2''\text{-}5''}$ = 2.9, $J_{2''\text{-}4''}$ = 1.3), 7.66 (dd, 1H, $C^{5''}$**H**, $J_{5''\text{-}4''}$ = 4.9, $J_{5''\text{-}2''}$ = 2.9), 7.57 (m, 2H, $C^{2'}$**H**), 7.36 (dd, 1H, $C^{4''}$**H**, $J_{4''\text{-}5''}$ = 4.9, $J_{4''\text{-}2''}$ = 1.3), 7.31 (d, 1H, $C^{1'}$-CH=**CH**, $J_{H\text{-}C=C\text{-}H}$ = 15.6), 6.76 (m, 2H, $C^{3'}$**H**), 3.49-3.55 (m, 2H, C**H_2**-OH), 3.09, 2.95-3.05, 2.89 (3x m, 4H, C^5**H_2** and C^8**H_2**), 2.97 (s, 6H, N-(C**H_3**)$_2$), 2.34, 2.10, 1.57-1.67 (3x m, 4H, C^4**H_2** and C^9**H_2**), 0.95 (m, 1H, C^2**H**), 0.71-0.78 (m, 2H, C^1**H** and C^3**H**).

^{13}C NMR (150.9 MHz, DMSO-d_6): δ 154.9 (C^6-**C**-N and C^7-**C**-N), 150.8 (**$C^{4'}$**), 139.2 (**C^6**), 138.8 (C^7 and $C^{3''}$), 135.5 ($C^{1'}$-**C**H=CH), 128.8 (**$C^{4''}$**), 128.6 (**$C^{2'}$**), 125.8 ($C^{5''}$), 125.4 ($C^{2''}$), 124.4 (**$C^{1'}$**), 116.6 ($C^{1'}$-CH=**C**H), 112.1 ($C^{3'}$), 57.1 (**C**H_2-OH), 39.8 (N-(**C**H_3)$_2$), 27.4 (**C^5** or **C^8**), 25.5 (**C^5** or **C^8**), 23.1 (**C^4** or **C^9**), 22.2 (**C^4** or **C^9**), 21.8 (C^2), 18.4 (**C^1** and **C^3**) .

HRMS (ESI): m/z calcd. for $C_{26}H_{29}N_3OS$ $[MH]^+$ 432.2104, found 432.2102.

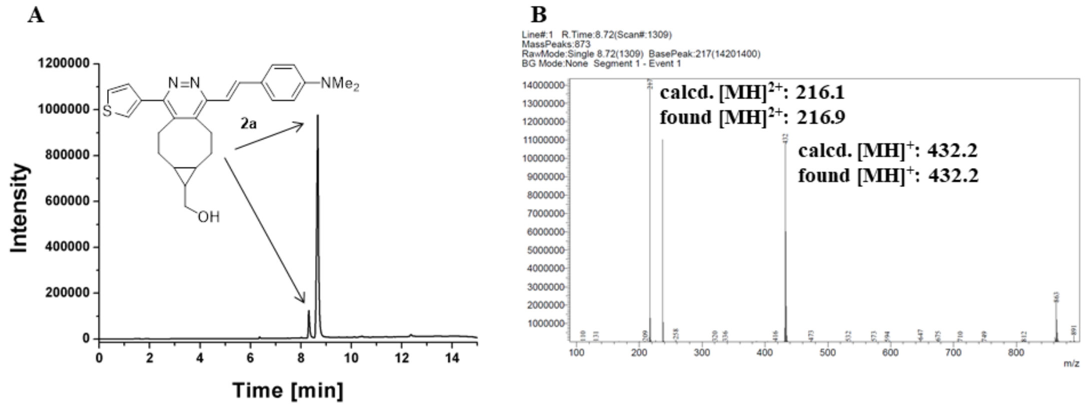

Figure S1. HPLC chromatogram and low-resolution mass spectrum of click product **2a** measured in CH_3CN/H_2O 1:1 at room temperature (solvent A: H_2O + 0.05% HCOOH; solvent B: CH_3CN + 0.05% HCOOH; gradient: 5% B → 95% B in 9 min, then 95% B for 2 min and back to 5% B; column: Luna® C18(2) column (3µm, 100A, 100 x 4.6 mm) at a flow rate of 1 mL/min).

Photophysical properties of the click products of BCN with tetrazines 1a-1e

Conditions for the following absorption and emission measurements:

A 1 mM solution of the respective tetrazine in DMSO was mixed with a 50 mM solution of BCN in the indicated solvent (CH_3CN, CH_3CN/H_2O 1:1, $CHCl_3$, acetone, dioxane, *i*PrOH or MeOH) and further diluted with the corresponding solvent to give a final tetrazine concentration of 500 µM using 10 equiv of BCN. The reaction mixtures were incubated at room temperature in the dark for 19 h and then measured by HPLC-MS (Figure S2) to verify the formation of the corresponding click products.

HPLC-MS measurements were performed on a Luna® C18(2) column (3µm, 100A, 100 x 4.6 mm) using a linear gradient of CH_3CN + 0.05% HCOOH (5→95% in 9 min) in H_2O + 0.05% HCOOH at a flow rate of 1.0 mL/min. All low-resolution masses found during these measurements are summarized in Table S1. These stock solutions were further diluted to a tetrazine concentration of 25 µM for absorption measurements and to a concentration of 2.5 µM for emission measurements.

Table S1. Calculated and observed masses of click products **2a-2e**[a].

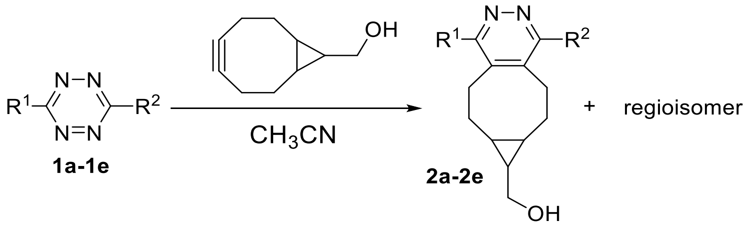

Click-product	R1	R2	M [g/mol]	
			calcd.	found
**2a**	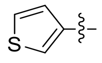	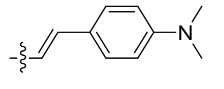	432.2	432.2
**2b**	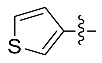	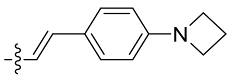	444.2	444.2
**2c**	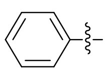	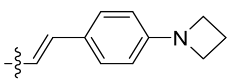	438.3	438.3
**2d**	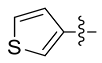	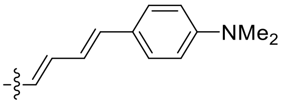	458.2	458.2
**2e**	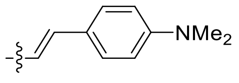		495.3	495.3

a) All reactions were performed in CH_3CN at room temperature using an excess of BCN and further diluted in 1:1 ratio with CH_3CN for HPLC-MS measurements.

HPLC chromatograms and low-resolution mass spectra of click products 2a-2e

A

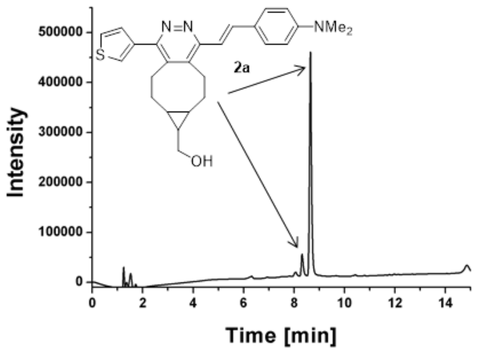

NMe2
2a
OH
Intensity
Time [min]

B

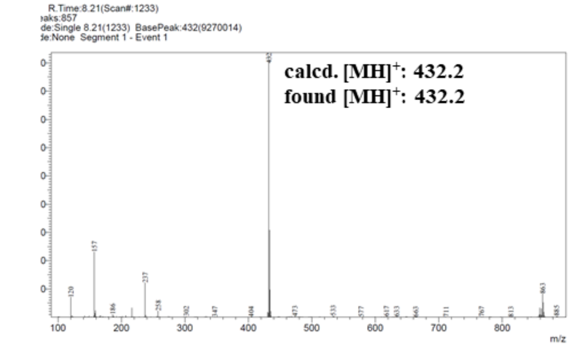

calcd. [MH]+: 432.2
found [MH]+: 432.2
m/z

A

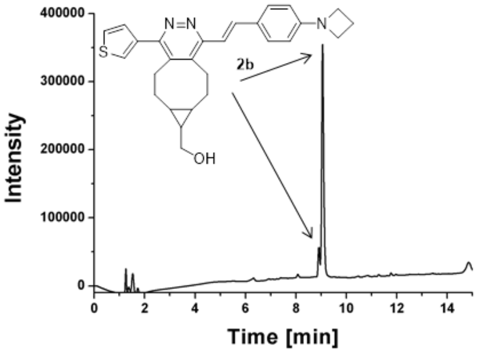

2b
OH
Intensity
Time [min]

B

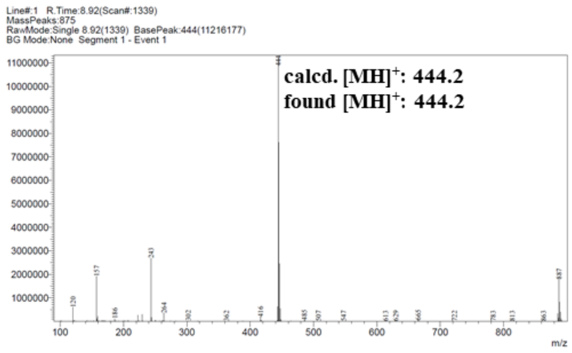

calcd. [MH]+: 444.2
found [MH]+: 444.2
m/z

A

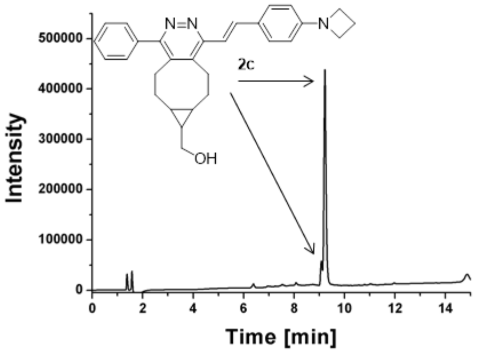

2c
OH
Intensity
Time [min]

B

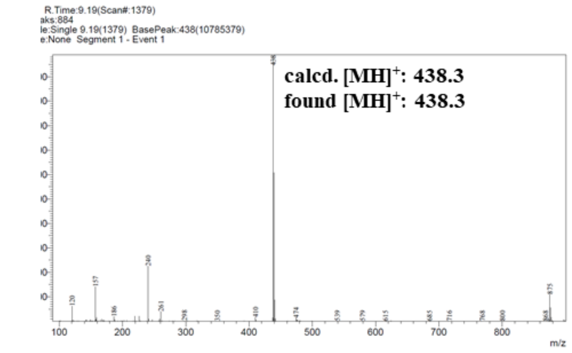

calcd. [MH]+: 438.3
found [MH]+: 438.3
m/z

A

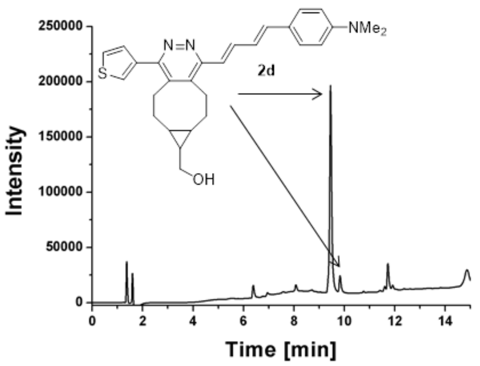

NMe2
2d
OH
Intensity
Time [min]

B

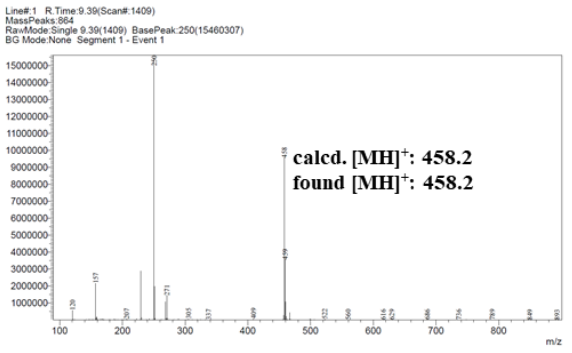

calcd. [MH]+: 458.2
found [MH]+: 458.2
m/z

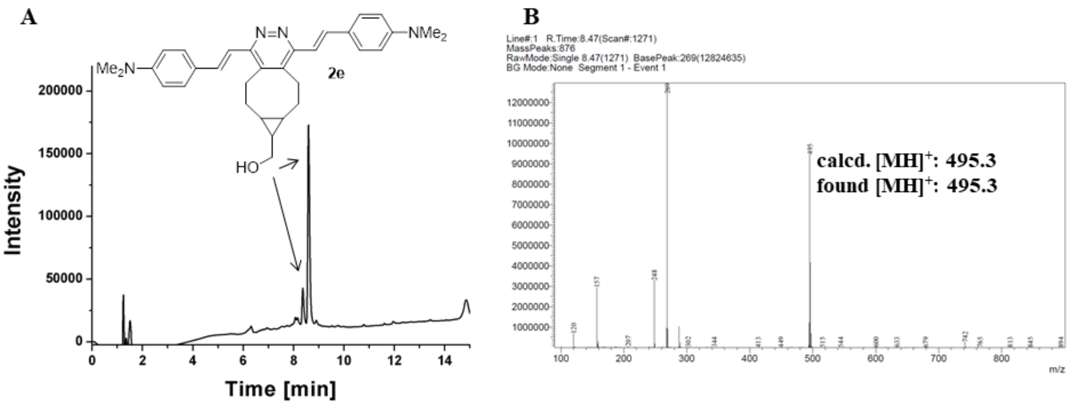

Figure S2. HPLC chromatograms (A's) and low-resolution mass spectra (B's) of click products **2a-2e** (solvent A: H_2O + 0.05% HCOOH; solvent B: CH_3CN + 0.05% HCOOH; gradient: 5% B → 95% B in 9 min, then 95% B for 2 min and back to 5% B; column: Luna® C18(2) column (3µm, 100A, 100 x 4.6 mm) at a flow rate of 1 mL/min).

Determination of fluorescence quantum yields

Quantum yields of click products were measured at room temperature (22 °C) in the indicated solvents (CH_3CN, CH_3CN/H_2O 1:1, $CHCl_3$, acetone, dioxane, *i*PrOH or MeOH) at 2.5 µM final concentration using a 1 cm quartz cuvette and quinine sulfate (solution in 0.5 M H_2SO_4) as reference (ϕ_{QS} = 0.55). The settings were as follows: Excitation wavelength 380 nm, slit 3.0 nm; Emission 400-700 nm, increment 1.0 nm, slit 3.0 nm and data algebra formula S1c/R1c. The fluorescence quantum yields were calculated using the following equation:

$$\phi_{sample} = \phi_{ref} \frac{F_{sample}}{F_{ref}} \times \frac{(1-10^{-abs})_{ref}}{(1-10^{-abs})_{sample}} \times \frac{n^2_{sample}}{n^2_{ref}}$$

Where:

$\boldsymbol{\phi_{ref}}$ is 0.55 (fluorescence quantum yield of quinine sulfate in 0.5 M H_2SO_4)[5]

F are the integrated intensities (areas) of the standard and the sample fluorescence spectra (integrals calculated using OriginPro software)

abs are the absorptions of standard and sample at the excitation wavelength (380 nm)

n are the refractive indices for standard (0.5 M H_2SO_4: 1.333) and sample solution (CH_3CN: 1.3404, CH_3CN/H_2O (1:1): 1.3478)[6]

Photophysical properties and absorption and emission spectra measured in CH_3CN

Table S2. Summary of photophysical properties of the click products of BCN with tetrazines **1a-1e** in CH_3CN[a].

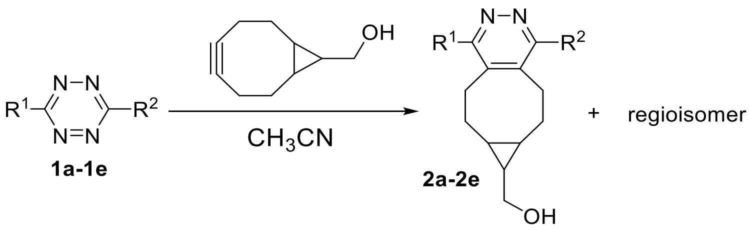

Click-product	R^1	R^2	$\lambda_{Abs}/\lambda_{Em}$ [b] [nm]	Stokes shift [nm]	ϕ [c]	ε_{max} ($\times10^3$ M^{-1} cm^{-1})	Fl. intensity increase [d]
**2a**	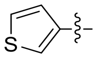	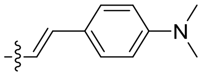	376/506	130	0.014	21.3	100-fold
**2b**	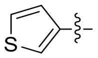	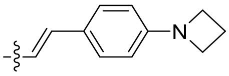	370/513	143	0.010	20.8	185-fold
**2c**	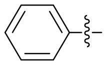	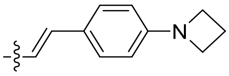	369/508	139	0.011	23.6	330-fold
**2d**	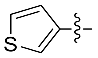	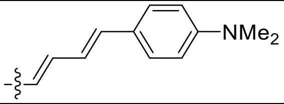	392/562	170	0.038	11.0	10-fold
**2e**	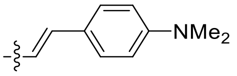		397/529	132	0.134	13.8	900-fold

a) All reactions were performed in CH_3CN at room temperature using an excess of BCN, b) absorption and emission (at 380 nm excitation) maxima were measured in CH_3CN at room temperature, c) quantum yields were determined by using quinine sulfate in 0.5 M H_2SO_4 as standard (ϕ = 0.55), d) calculated as the integral of the fluorescence of the click product divided by the integral of the fluorescence of the starting tetrazine.

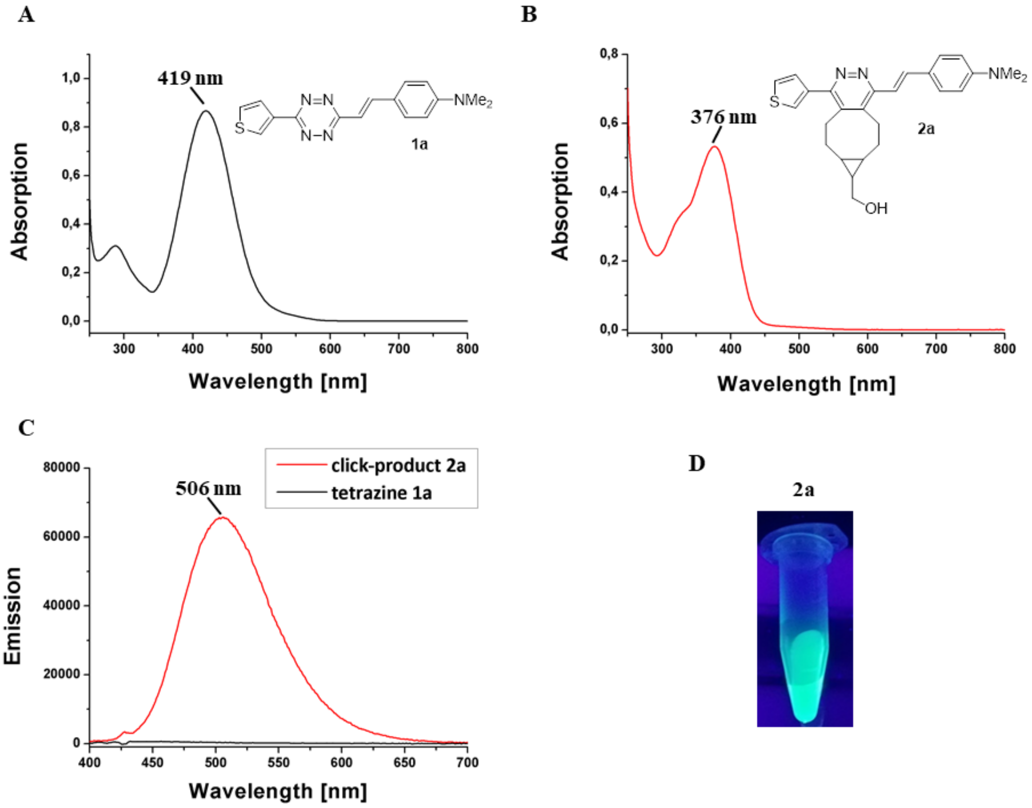

Figure S3. Absorption and emission spectra of tetrazine **1a** (A and C) and click product **2a** (B and C) and fluorescence picture of click product **2a** (D). All spectra were measured in CH_3CN at room temperature using a tetrazine concentration of 25 µM for absorption measurements and 2.5 µM for emission measurements.

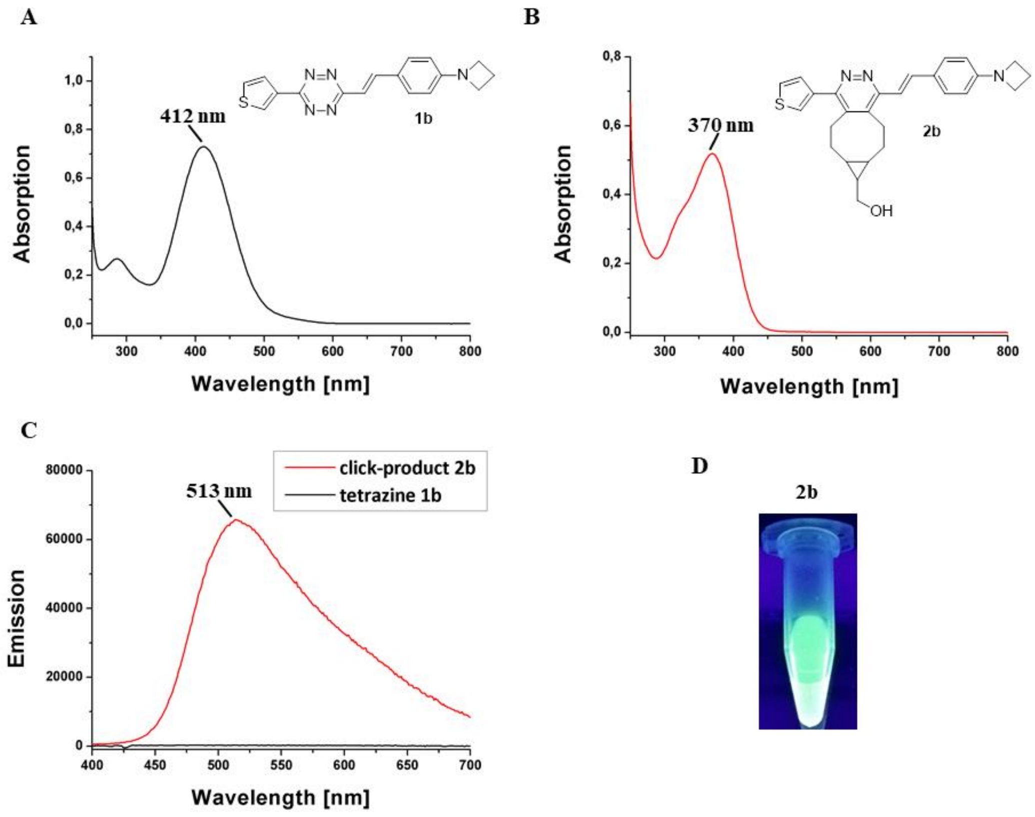

Figure S4. Absorption and emission spectra of tetrazine **1b** (A and C) and click product **2b** (B and C) and fluorescence picture of click product **2b** (D). All spectra were measured in CH_3CN at room temperature using a tetrazine concentration of 25 µM for absorption measurements and 2.5 µM for emission measurements.

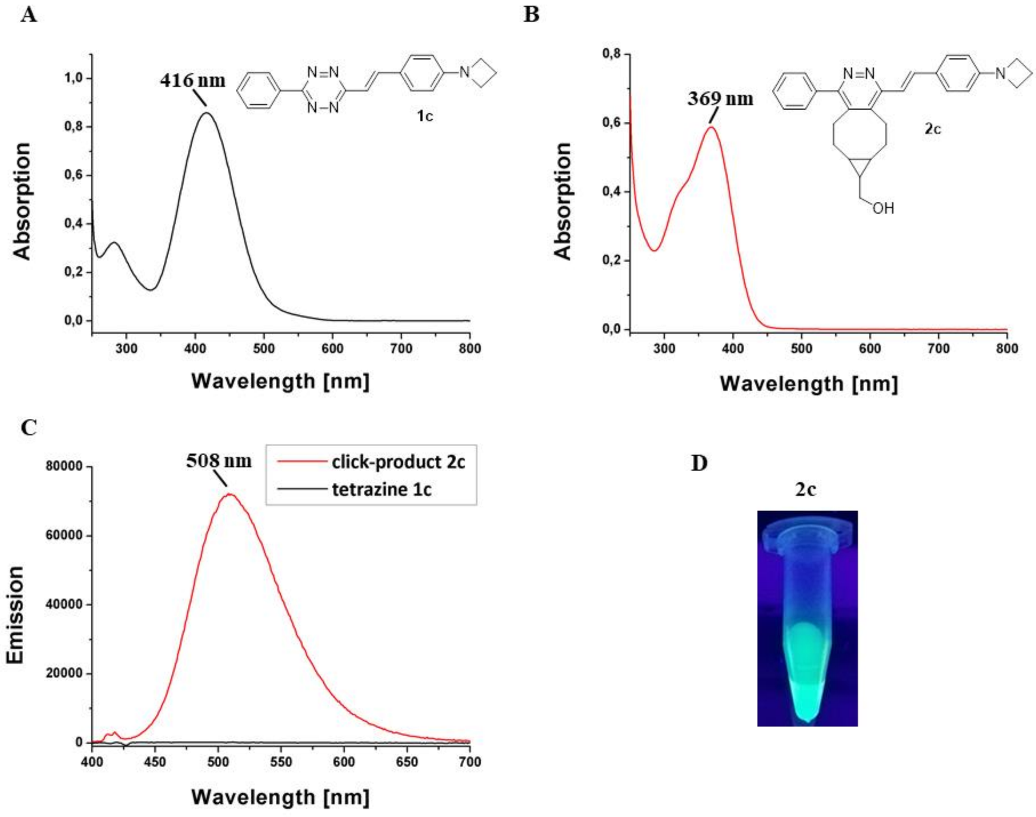

Figure S5. Absorption and emission spectra of tetrazine **1c** (A and C) and click product **2c** (B and C) and fluorescence picture of click product **2c** (D). All spectra were measured in CH_3CN at room temperature using a tetrazine concentration of 25 µM for absorption measurements and 2.5 µM for emission measurements.

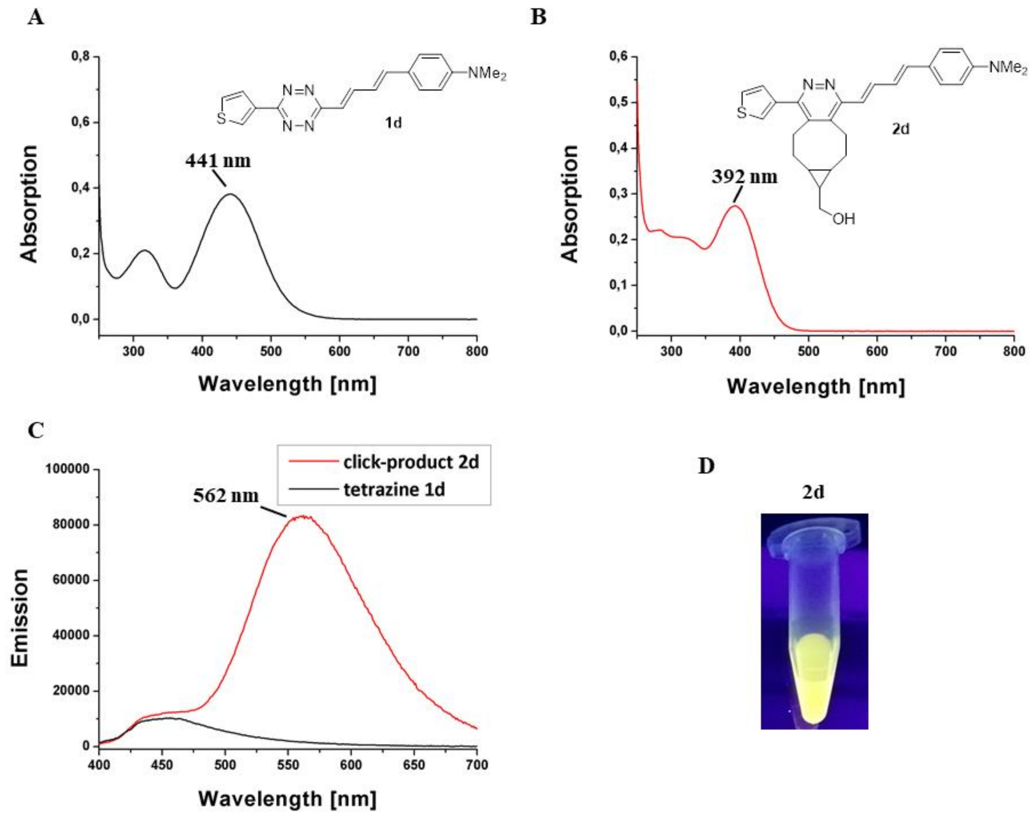

Figure S6. Absorption and emission spectra of tetrazine **1d** (A and C) and click product **2d** (B and C) and fluorescence picture of click product **2d** (D). All spectra were measured in CH_3CN at room temperature using a tetrazine concentration of 25 µM for absorption measurements and 2.5 µM for emission measurements.

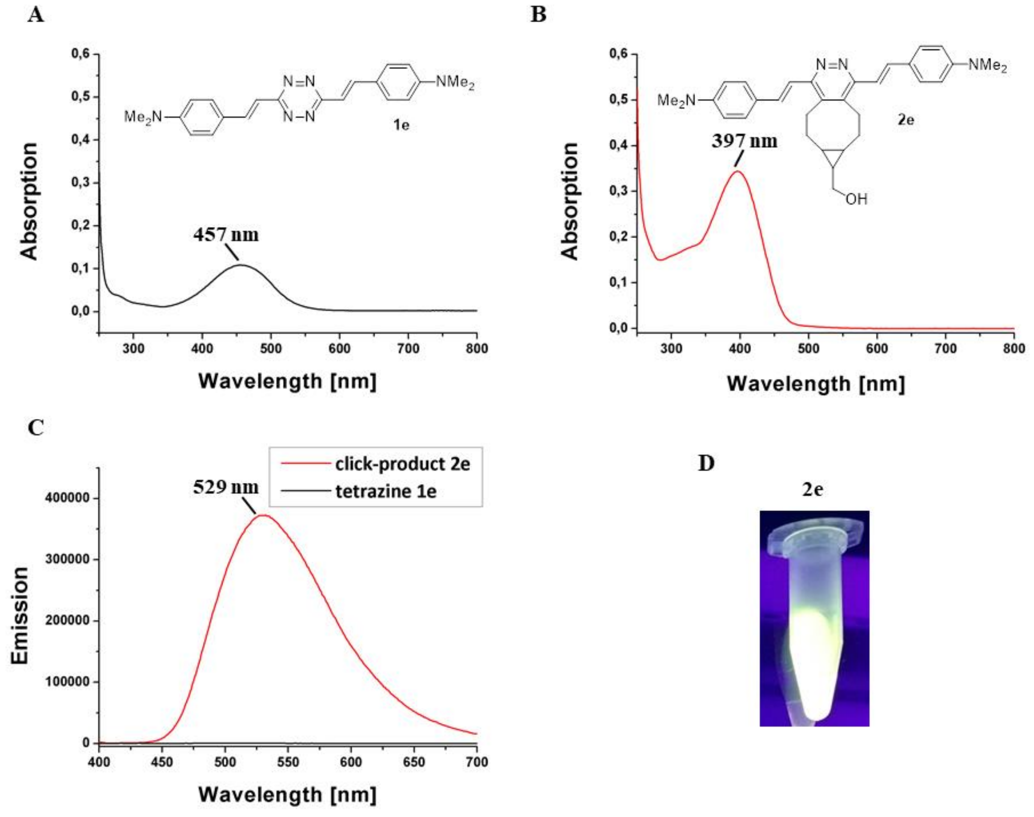

Figure S7. Absorption and emission spectra of tetrazine **1e** (A and C) and click product **2e** (B and C) and fluorescence picture of click product **2e** (D). All spectra were measured in CH_3CN at room temperature using a tetrazine concentration of 25 µM for absorption measurements and 2.5 µM for emission measurements.

Photophysical properties and absorption and emission spectra measured in CH_3CN/H_2O 1:1

Table S3. Photophysical properties of the click products of BCN with tetrazines **1a-1e** in CH_3CN/H_2O 1:1[a].

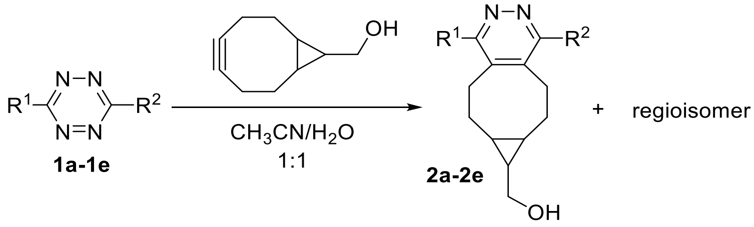

Click-product	R^1	R^2	$\lambda_{Abs}/\lambda_{Em}$[b] [nm]	Stokes shift [nm]	ϕ[c]	ε_{max} ($\times 10^3$ M^{-1} cm^{-1})	Fl. intensity increase[d]
**2a**	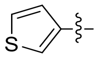	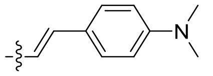	378/531	153	0.010	21.2	210-fold
**2b**	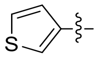	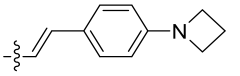	365/537	172	0.008	20.4	285-fold
**2c**	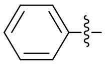	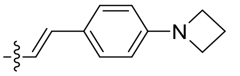	365/538	173	0.008	23.0	120-fold
**2d**	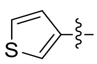	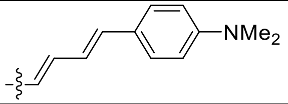	392/600	208	0.033	11.2	20-fold
**2e**	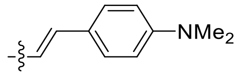		-[e]/553	-	0.011	-	20-fold
**2e**	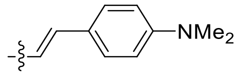		396/558[f]	162[f]	0.019[f]	2.2[f]	70-fold[f]

a) All reactions were performed in CH_3CN/H_2O 1:1 at room temperature using an excess of BCN, b) absorption and emission (at 380 nm excitation) maxima were measured in CH_3CN/H_2O 1:1 at room temperature, c) quantum yields were determined by using quinine sulfate in 0.5 M H_2SO_4 as standard (ϕ = 0.55), d) calculated as the integral of the fluorescence of the click product divided by the integral of the fluorescence of the starting tetrazine, e) no absorption maxima detectable in CH_3CN/H_2O 1:1, f) reaction and measurements were performed in CH_3CN/PBS 1:1 at room temperature.

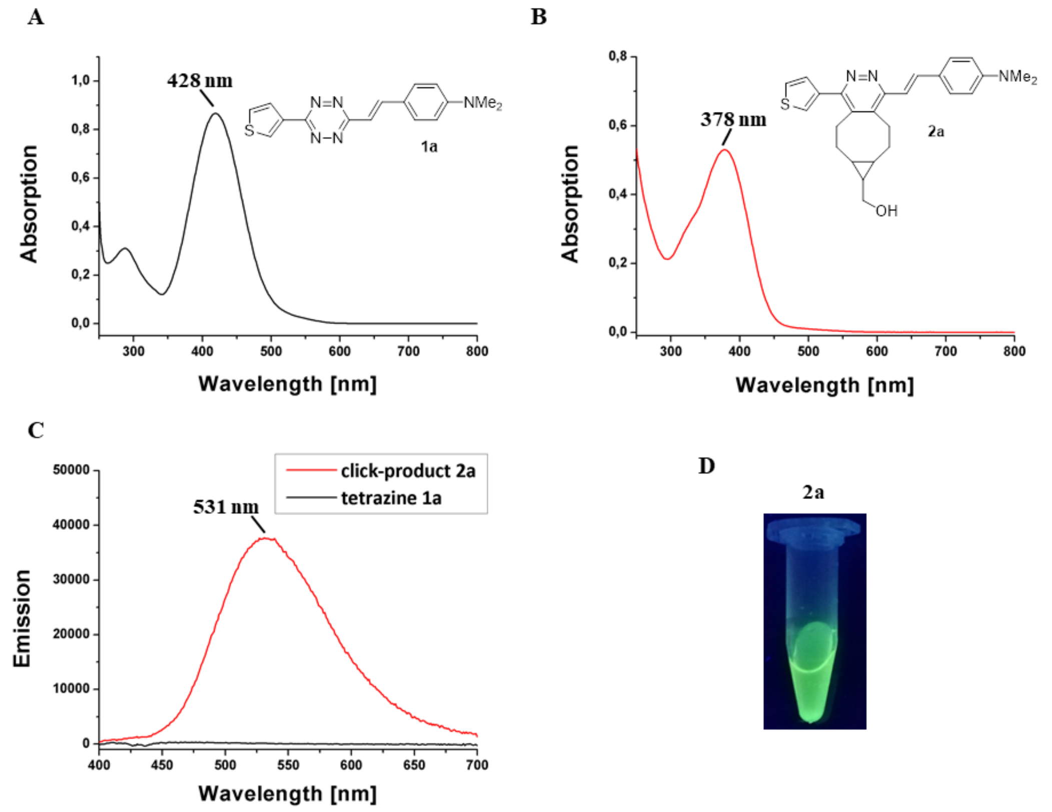

Figure S8. Absorption and emission spectra of tetrazine **1a** (A and C) and click product **2a** (B and C) and fluorescence picture of click product **2a** (D). All spectra were measured in CH_3CN/H_2O 1:1 at room temperature using a tetrazine concentration of 25 µM for absorption measurements and 2.5 µM for emission measurements.

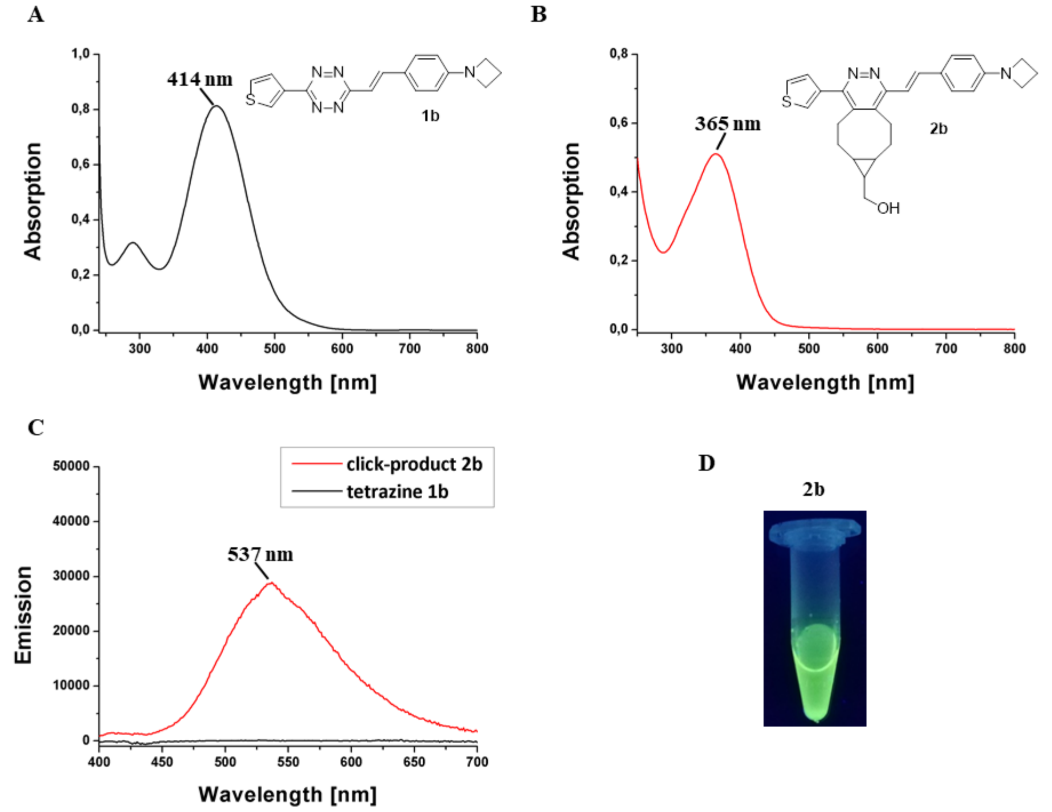

Figure S9. Absorption and emission spectra of tetrazine **1b** (A and C) and click product **2b** (B and C) and fluorescence picture of click product **2b** (D). All spectra were measured in CH_3CN/H_2O 1:1 at room temperature using a tetrazine concentration of 25 µM for absorption measurements and 2.5 µM for emission measurements.

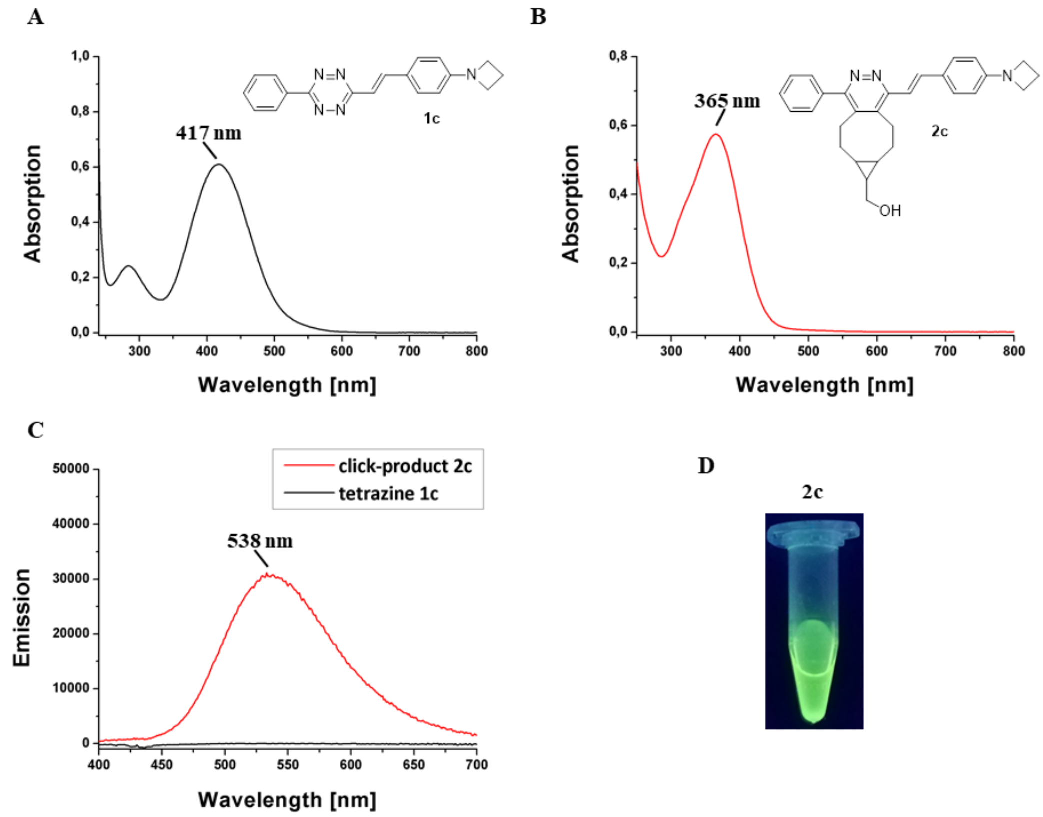

Figure S10. Absorption and emission spectra of tetrazine **1c** (A and C) and click product **2c** (B and C) and fluorescence picture of click product **2c** (D). All spectra were measured in CH_3CN/H_2O 1:1 at room temperature using a tetrazine concentration of 25 µM for absorption measurements and 2.5 µM for emission measurements.

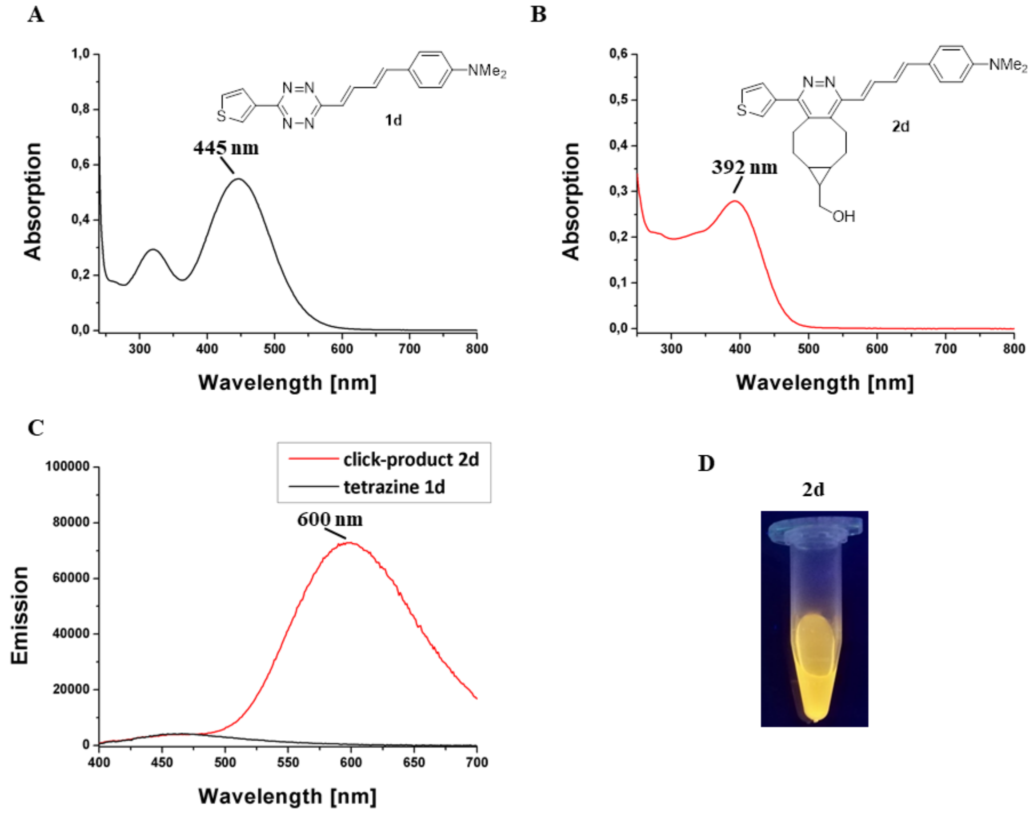

Figure S11. Absorption and emission spectra of tetrazine **1d** (A and C) and click product **2d** (B and C) and fluorescence picture of click product **2d** (D). All spectra were measured in CH_3CN/H_2O 1:1 at room temperature using a tetrazine concentration of 25 µM for absorption measurements and 2.5 µM for emission measurements.

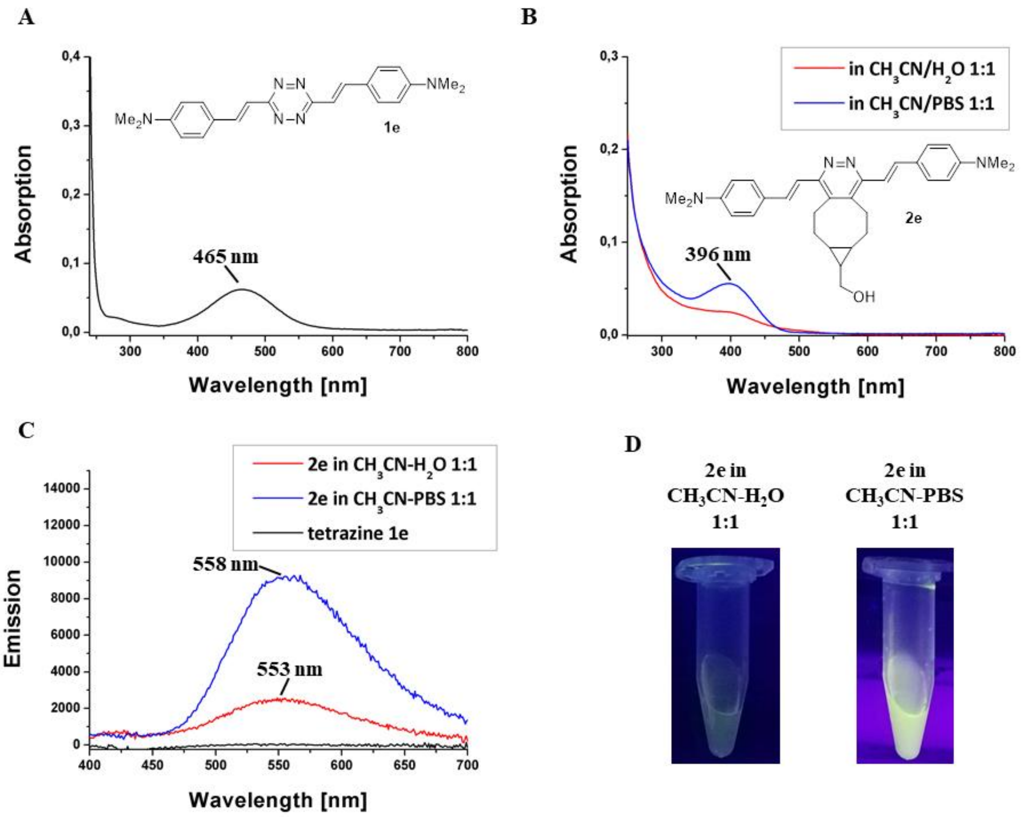

Figure S12. Absorption and emission spectra of tetrazine **1e** (A and C) and click product **2e** (B and C) and fluorescence pictures of click product **2e** (D). All spectra were measured in CH_3CN/H_2O 1:1 or CH_3CN/PBS 1:1 at room temperature using a tetrazine concentration of 25 µM for absorption measurements and 2.5 µM for emission measurements.

Photophysical properties, absorption and emission spectra of 2a in different solvents

Table S4. Photophysical properties of click product **2a** in different solvents[a].

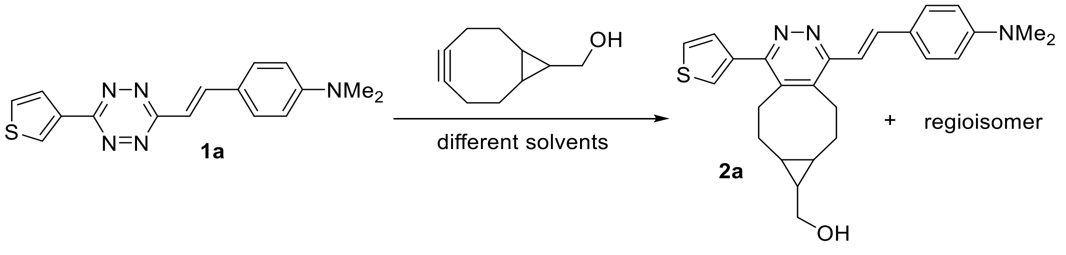

solvent	$\lambda_{Abs}/\lambda_{Em}$ [b] [nm]	Stokes shift [nm]	ϕ [c]	ε_{max} ($\times 10^3$ $M^{-1}cm^{-1}$)	Fl. intensity increase [d]
$CHCl_3$	383/476	93	0.004	21.2	50-fold
Acetone	377/493	116	0.008	25.0	45-fold
Dioxane	377/463	86	0.002	26.5	13-fold
*i*PrOH	381/499	118	0.010	22.5	40-fold
MeOH	381/518	137	0.010	25.6	55-fold
CH_3CN	376/506	130	0.014	21.3	100-fold
CH_3CN/H_2O 1:1	378/531	153	0.010	21.2	210-fold

a) The click-reaction was performed in the indicated solvents ($CHCl_3$, acetone, dioxane, iPrOH, MeOH, CH_3CN or CH_3CN/H_2O 1:1) at room temperature using an excess of BCN, b) absorption and emission (at 380 nm excitation) maxima were measured in the corresponding solvent at room temperature, c) quantum yields were determined by using quinine sulfate in 0.5 M H_2SO_4 as standard (ϕ = 0.55), d) calculated as the integral of the fluorescence of the click product divided by the integral of the fluorescence of the starting tetrazine.

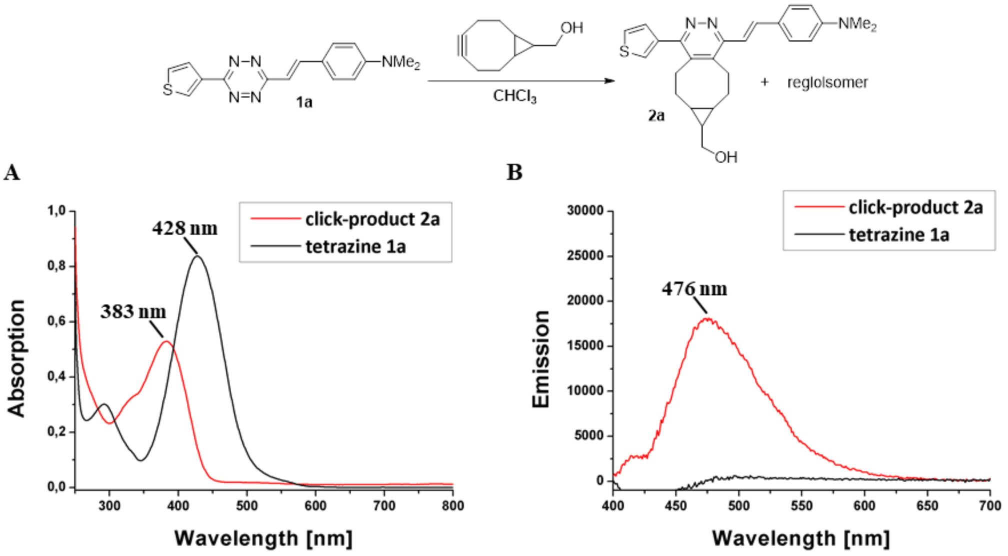

Figure S13. Absorption (A) and emission (B) spectra of tetrazine **1a** and click product **2a** in $CHCl_3$. All spectra were measured at room temperature using a tetrazine concentration of 25 µM for absorption measurements and 2.5 µM for emission measurements.

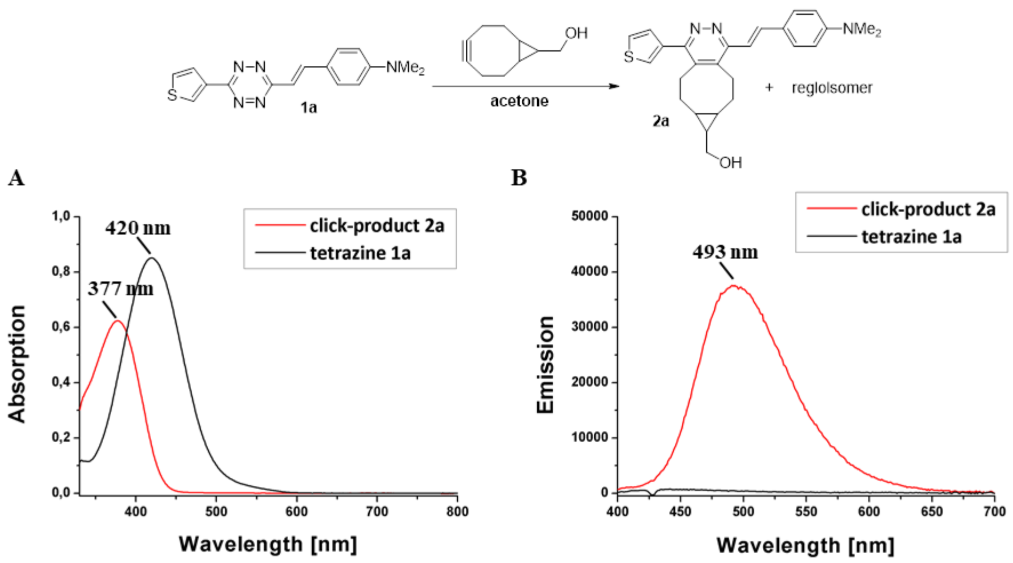

Figure S14. Absorption (A) and emission (B) spectra of tetrazine **1a** and click product **2a** in acetone. All spectra were measured at room temperature using a tetrazine concentration of 25 µM for absorption measurements and 2.5 µM for emission measurements.

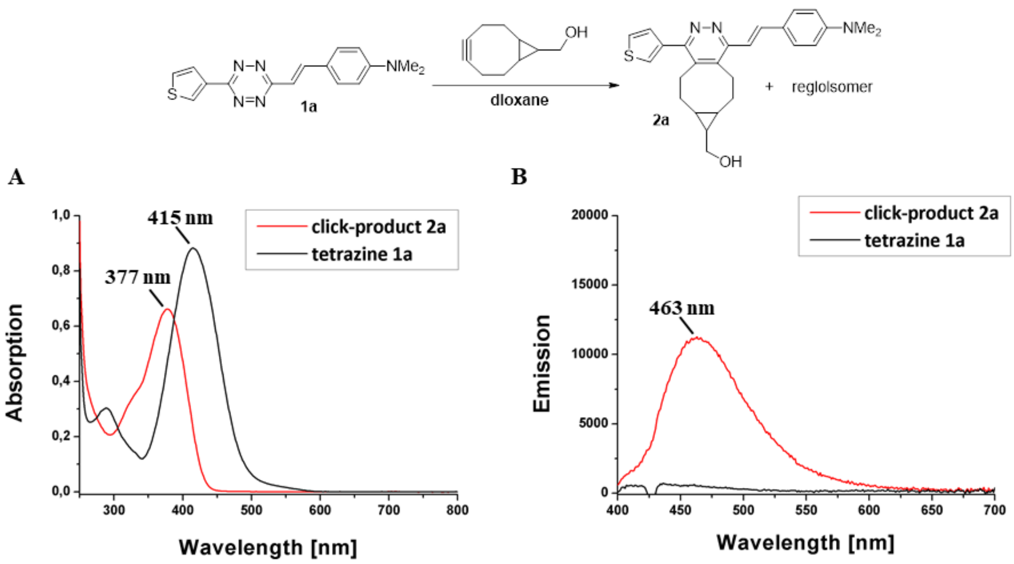

Figure S15. Absorption (A) and emission (B) spectra of tetrazine **1a** and click product **2a** in dioxane. All spectra were measured at room temperature using a tetrazine concentration of 25 µM for absorption measurements and 2.5 µM for emission measurements.

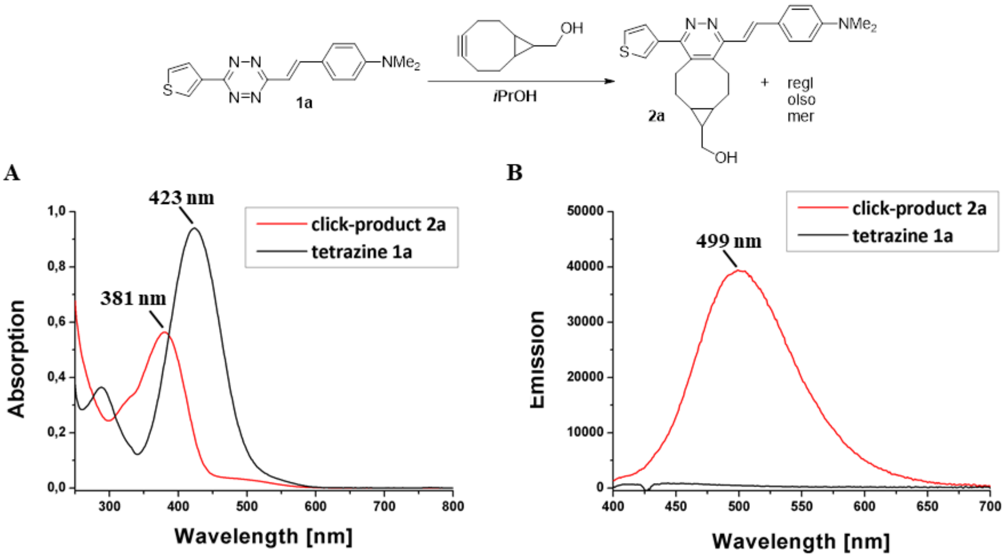

Figure S16. Absorption (A) and emission (B) spectra of tetrazine **1a** and click product **2a** in *i*PrOH. All spectra were measured at room temperature using a tetrazine concentration of 25 µM for absorption measurements and 2.5 µM for emission measurements.

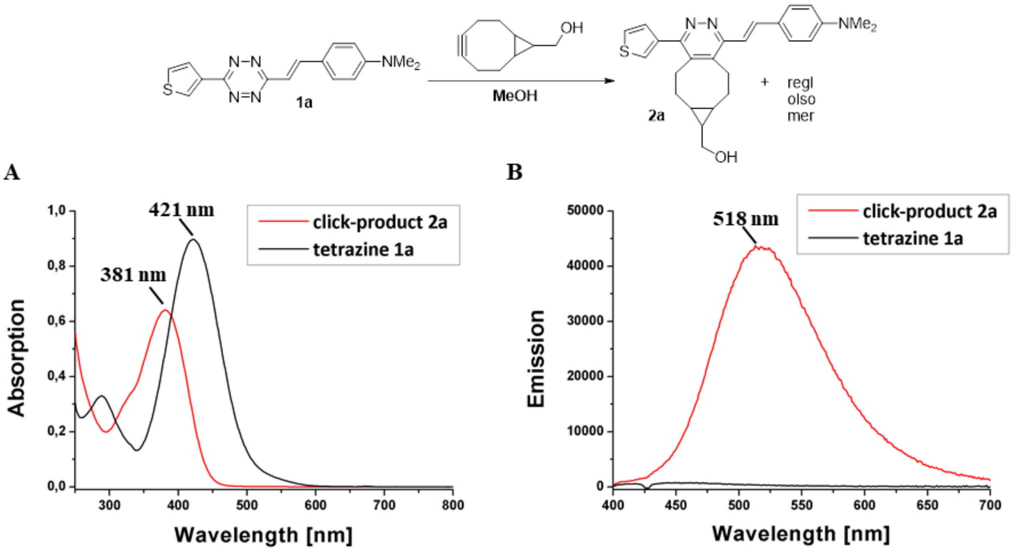

Figure S17. Absorption (A) and emission (B) spectra of tetrazine **1a** and click product **2a** in MeOH. All spectra were measured at room temperature using a tetrazine concentration of 25 µM for absorption measurements and 2.5 µM for emission measurements.

Determination of second-order rate constants

Second-order rate constants of the reactions of bicyclononyne (BCN) with 1,2,4,5-tetrazines **1a** and **1d** were determined by following the decay in the concentration of the starting tetrazine over time. The rate constants were calculated from the pseudo first order rate constants of the concentration decrease measured at different BCN concentrations (10 equiv, 12.5 equiv and 15 equiv) by UV/VIS spectroscopy. The measurements were performed in CH_3CN/H_2O 1:1 at room temperature using a final tetrazine concentration of 12.5 µM. All measurements were performed at least three times.

Conditions: A 50 µM solution of the respective tetrazine in CH_3CN/H_2O 1:1 containing 5% of DMSO was mixed with a 500 µM solution of BCN in CH_3CN/H_2O 1:1. The mixture was further diluted with CH_3CN/H_2O 1:1 to give a final tetrazine concentration of 12.5 µM and was immediately measured on the UV/VIS spectrophotometer.

The time-dependent measurements were performed at the corresponding absorption maxima of the tetrazines used, which were determined by UV/VIS spectroscopy before the measurement (Table S5). The measured intensity of the absorption was plotted against time. Fitting the curves with single exponential equation ($y = y_0 + Ae^{-k/t}$) provided the observed rate constants. Finally, the observed rate constants were plotted against the concentration of BCN in order to obtain the second order rate constants (Table S5) from the slope of the resulting plot.

Table S5. Absorption maxima of 1,2,4,5-tetrazines used during kinetic measurements and the corresponding second-order rate constants k_2 of the reactions with BCN.

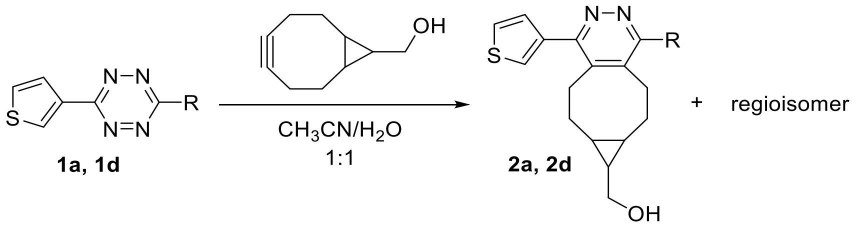

Tetrazine	λ_{Abs} [nm][a]	k_2 [M^{-1} s^{-1}][b]
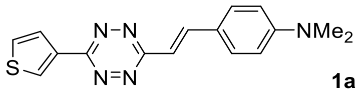 **1a**	428	1.4 ± 0.2
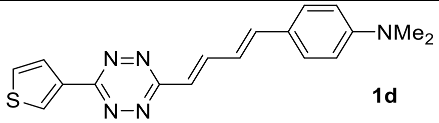 **1d**	445	1.5 ± 0.1

a) Absorption maxima of the starting tetrazines were determined in CH_3CN/H_2O 1:1 at room temperature using a 25 µM solution of the respective tetrazine, b) second-order rate constants were determined from pseudo first order rate constants measured in CH_3CN/H_2O 1:1 at room temperature using three different BCN concentrations (10, 12.5 and 15 equiv).

Fluorogenic cell labeling

HeLa cells fixed with methanol were rehydrated in 0.05% Tween in PBS and incubated with 100 µM BCN-NHS for 1 hour. The cells were then washed three-times with 0.05% Tween in PBS, incubated with 20 µM tetrazines for 4 hours and the nucleus was stained using commercially available DRAQ5. The cells were again washed three-times with 0.05% Tween in PBS and inspected on confocal microscope.

ConA-BCN preparation

100 µl of 5 mg/ml solution of concanavalin A (4.7 nmol of tetramer) in 1 M NaCl, 50 mM HEPES-NaOH (pH 8.3), 3 mM $CaCl_2$, 3 mM $MnCl_2$ was combined with 2.3 µL of 10 mM NHS BCN ester (5× molar excess of active ester dissolved in dry DMSO). Reaction was incubated at room temperature for 1 h with constant shaking. After one hour, 10 µL of 1 M Tris-HCl (pH 6.8) was added to neutralize the remaining NHS ester and incubated for another 10 min. at room temperature. BCN excess was removed by desalting using Zeba (Thermo) spin columns into 1 M NaCl, 20 mM Tris-HCl (pH 6.8), 3 mM $CaCl_2$, 3 mM $MnCl_2$.

Live cell labeling

$2x10^4$ HeLa (or U2OS) cells were seeded on glass bottom 96 well plate (Cellvis) one day prior experiment. The cells were incubated with 5 µM BCN-TPP for 15 min, washed 3x with medium and incubated with 20 µM **1d** for 45 min. Medium was replaced with Leibowitz's L15 containing 10 nM mitotracker deep red and the cells were incubated for 15 min. Pictures were taken using Zeiss confocal microscope equipped with 40x C-Apochromat 40x/1.20 W Korr FCS M27 objective. Set-up: click product: excitation at 405 nm, emission at 508-604 nm window, laser intensity 1%. Mitotracker: excitation at 633 nm, emission at 640-735 nm window, laser intensity 3%.

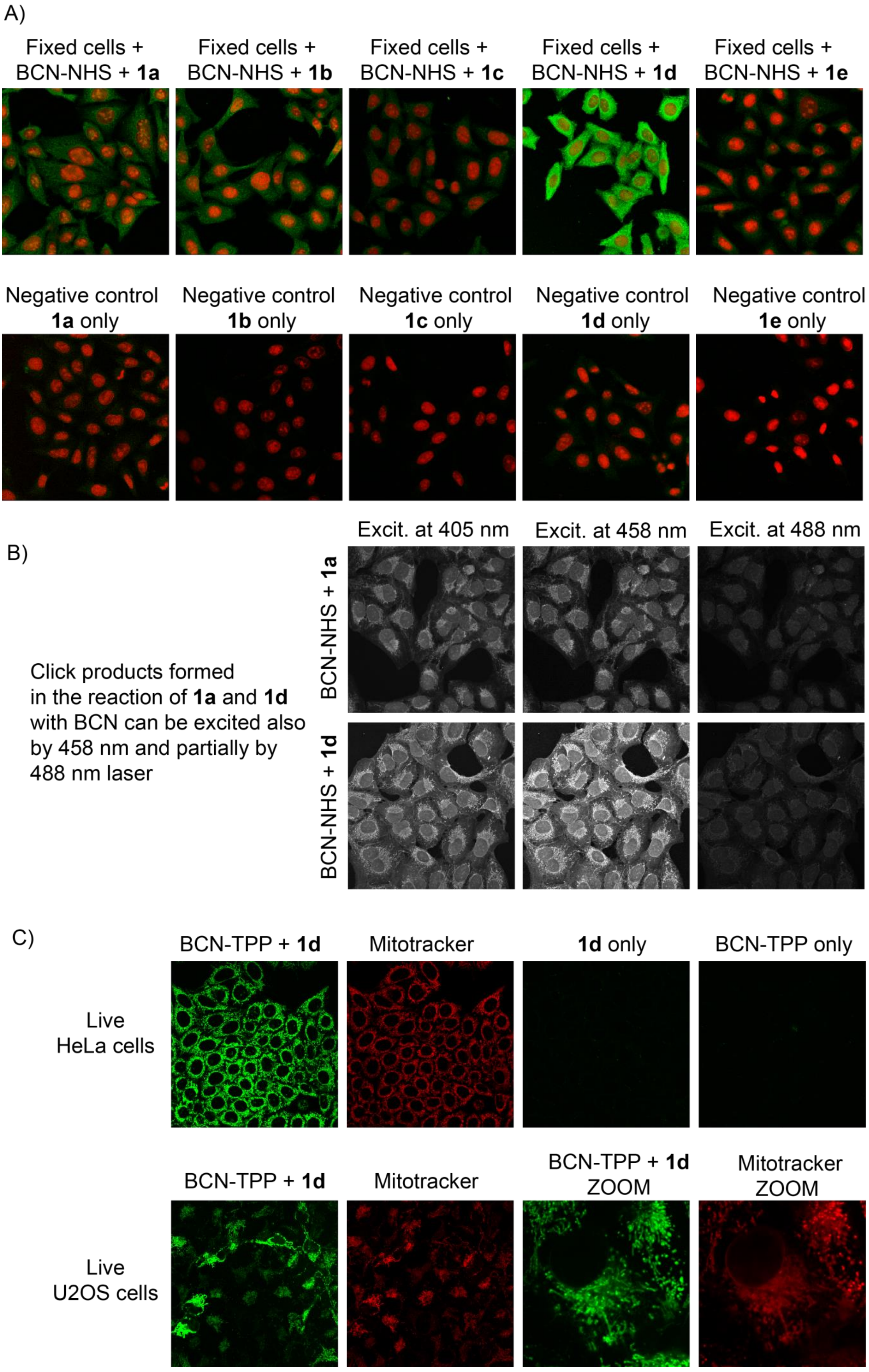
A)
Fixed cells + BCN-NHS + 1a
Fixed cells + BCN-NHS + 1b
Fixed cells + BCN-NHS + 1c
Fixed cells + BCN-NHS + 1d
Fixed cells + BCN-NHS + 1e
Negative control 1a only
Negative control 1b only
Negative control 1c only
Negative control 1d only
Negative control 1e only
B)
Excit. at 405 nm
Excit. at 458 nm
Excit. at 488 nm
BCN-NHS + 1a
BCN-NHS + 1d
Click products formed in the reaction of 1a and 1d with BCN can be excited also by 458 nm and partially by 488 nm laser
C)
BCN-TPP + 1d
Mitotracker
1d only
BCN-TPP only
Live HeLa cells
BCN-TPP + 1d
Mitotracker
BCN-TPP + 1d ZOOM
Mitotracker ZOOM
Live U2OS cells

Figure S18. Fluorogenic labeling of cells. A) Shown are confocal microscope images of HeLa cells modified with BCN-NHS active ester after addition of tetrazines **1a**-**1e**. Negative controls are HeLa cells treated only with tetrazines **1a**-**1e**. DRAQ5 was used as nuclear specific dye (Excitation: λ = 633 nm, Emission: λ = 643-703 nm window). Click product excitation λ = 458 nm, emission λ = 517-587 nm for **1a**, **1b**, **1c** and **1e** and λ = 543-639 window for **1d.** B) Click products **2a** and **2d** can be excited using both 405 nm and 458 nm laser. Emission: λ = 517-587 nm window for **1a** and λ = 543-639 window for **1d**. This experiment was performed on U2OS cancer cells. C) Live cell (HeLa and U2OS) labeling using BCN-TPP and **1d**.

Two-color fluorogenic labeling of segregated bilayer TG beads

The segregated bilayer Tentagel beads were prepared by following literature procedure.[7]
Briefly: Tentagel NH_2 resin (25 mg, 130 µm beads) was swollen in ddH_2O (2 mL) for 20 hours. After decantation the beads were briefly centrifuged, access water was pipetted off and the beads were briefly washed with DCM/Et_2O = 55/45 mixture (2 mL). A solution of BCN-NHS active ester (2.1 mg) in DCM/Et_2O = 55/45 mixture (2 mL) was added to the beads followed by DIPEA (2.5 µL) and the beads were rotated in a plastic tube for 30 min to modify the outer bead layer. The resin beads were then washed with DCM/Et_2O = 55/45 mixture, DCM and DMF (5-times). To modify the inner part of the beads a solution of TCO-NHS active ester (2.25 mg) in DMF (2 mL) was added to the beads followed by DIPEA (2.5 µL). The beads were rotated in a plastic tube for 1.5 hours and were then washed with DMF. For labeling with tetrazine **1a**: A small portion (ca 2-3 mg) of the beads was transferred to a plastic tube and CH_3CN/H_2O = 1/1 mixture (25 µL) was added followed by a solution of **1a** in DMSO (10 µL of 1 mM stock). After rotating the mixture for ca. 15-20 min the beads were washed with DMF and inspected on confocal microscope.
We have also prepared Tentagel beads modified only with BCN or TCO moiety respectively by the reaction of TG NH_2 beads with the BCN/TCO active esters (1 equiv.) in DMF using DIPEA (2 equiv.) as the base.
The BCN-NHS active ester is commercially available. TCO-NHS ester used in this experiment was prepared as previously described.[1] Structures of the NHS esters are shown below.

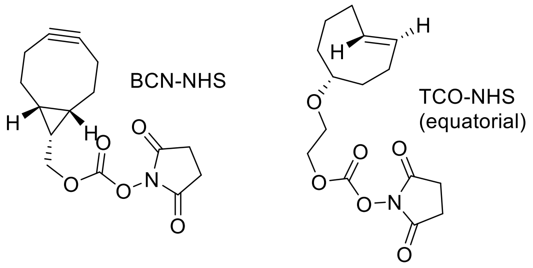

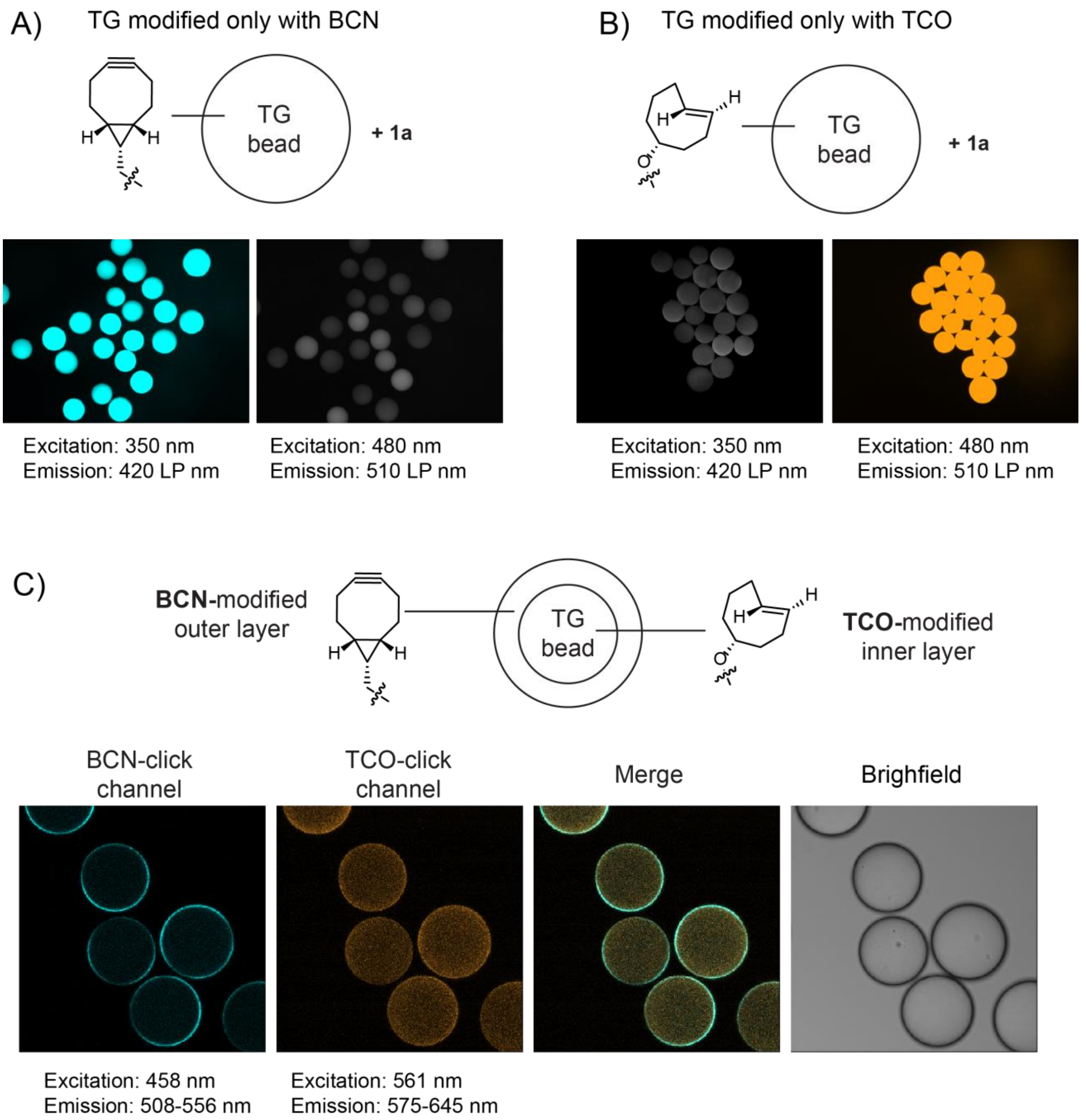

Figure S19. Fluorogenic labeling of TG beads modified with BCN and TCO dienophiles. A) Fluorescent stereomicroscope images of TG beads modified with BCN-NHS ester after addition of tetrazine **1a**. B) Fluorescent stereomicroscope images of TG beads modified with TCO-NHS ester after addition of tetrazine **1a**. C) Fluorescent confocal microscope images of segregated TG beads modified with BCN in the outer layer and TCO in the inner part after reaction with tetrazine **1a**. The images were captured by gray-scale camera and are in pseudocolors.

Toxicity studies

$2x10^4$ HeLa (or U2OS) cells were seeded on glass bottom 96 well plate (Cellvis) one day prior experiment. The cells were incubated with various concentrations of tetrazines **1e-1e** for 24 hours. The tetrazines showed no toxicity up to 50 µM concentration. Compound **1d** showed toxicity at 100 µM, the highest concentration tested. The toxicity was determined using XTT assay or by using crystal violet (Figure S20). The experiments were performed in triplicate.

Briefly: Cells were cultivated for 24h with indicated concentrations of compounds. After 24h 50 μL of XTT(+PMS) was added to cell medium. Absorbance (difference 450-620 nm) was measured 1h after addition of XTT.

XTT (Thermo) was dissolved in DMEM (high glucose no serum) to give concentration 1mg/ml, PMS (phenmetrazine sulfate, Sigma) was dissolved in PBS to final concentration 0.383 mg/ml (1.25mM) solutions were combined in ratio 50:1 and added to cell culture medium. Total absorbane was calculated as absorbance measured at 450 nm subtracted by absorbance value measured at 620 nm.

Crystal violet:
Cells grown on 96 well plate were fixed with 100μl of 100% methanol for 10 minutes, rinsed 2x with water and incubated with 50 μl of 0.1% crystal violet solution. After 15 minutes of incubation crystal violet was removed, cells were washed 2x with water. Crystal violet adsorbed on cells was dissolved in 50 μl of 100% methanol. Absorbance was measured at 595 nm using plate reader.

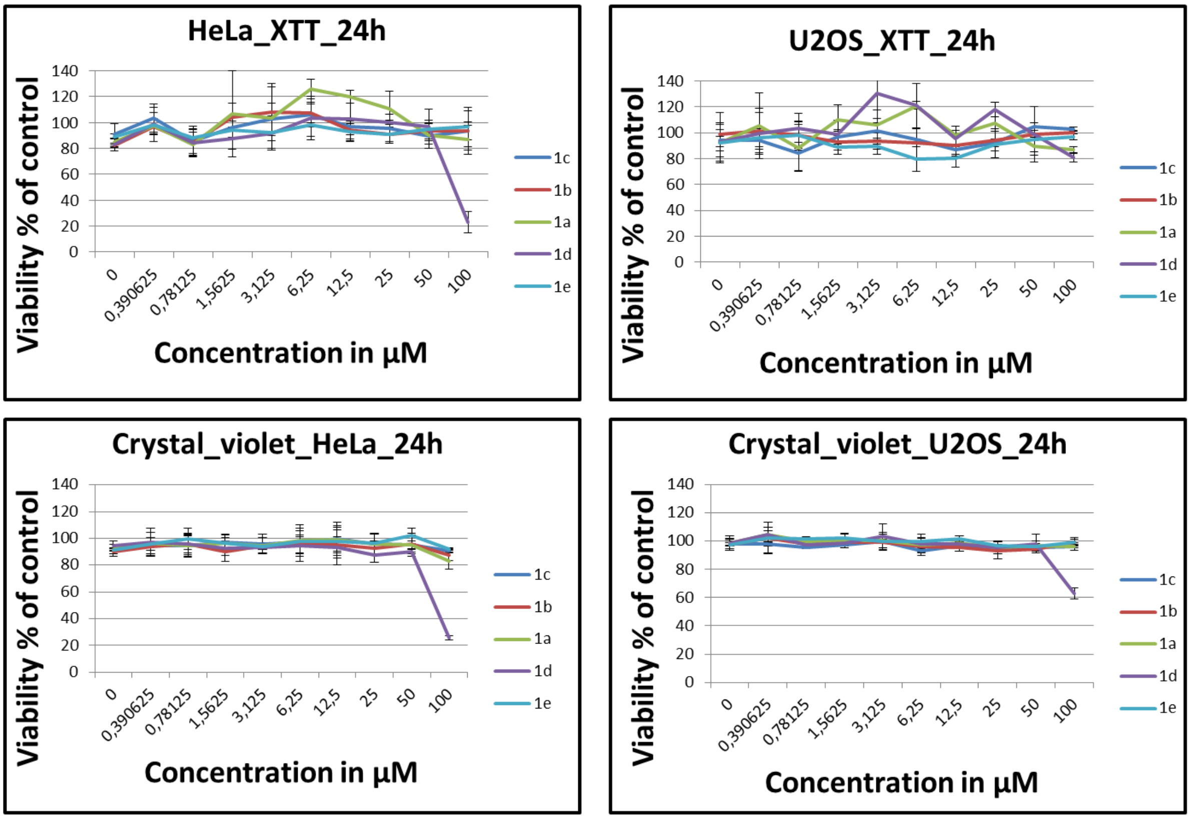

Figure S20. Toxicity of the tetrazines determined by XTT or crystal violet on HeLa or U2OS cells. Viability was calculated as % of control (DMSO).

Time-lapse labeling of BCN- and TCO-modified TG beads

TG beads modified with BCN or TCO (prepared as described above) were reacted with tetrazines **1a**, **1d** and **1e** as follows: To small portion of the beads (ca. 5 mg) was added CH_3CN/H_2O = 1/1 mixture (50 µL) and solution of the tetrazine in DMSO (10 µL of 1 mM stock). After 15 min, the reactions were inspected under UV-hand held lamp (365 nm) to confirm formation of the fluorophores. The beads were then washed twice with DMF, twice with CH_3CN/PBS= 1/1 mixture and finally were incubated at 37°C in 75 µL CH_3CN/PBS = 1/1 mixture for 24 hours. Small portion of the beads was pipetted off and the beads were inspected under fluorescence stereomicroscope at different time points (Leica M205 fluorescent stereomicroscope equipped with pE-300^{white} LED light source and DFC3000 G grayscale camera). Set-up on the microscope was as follows: UV: Ex. 350 nm, Em. 420 nm (long pass), intensity: 30%, gain: 8, exposure: 1.25 s. GFP: 480 nm, Em. 510 nm (long pass), intensity: 20%, gain: 8, exposure: 1.25 s. RFP: 546 nm, Em. 590 nm (long pass), intensity: 50%, gain: 8, exposure: 1.25 s.
The results are shown in Figure S21 below.

Figure S21. Fluorogenic labeling of TG beads modified with BCN and TCO dienophiles. Tautomeriztaion of the 4,5-dihydropyridazine formed in the reaction of the tetrazines with TCO and/or oxidation to the corresponding pyridazine leads to changes in the fluorescence over time. Based on our previous study (ChemBioChem, 2019, 20, 886-890) the half-life of the 4,5-dihydropyridazine is about 2 hours. This may vary depending on the structure of the tetrazine used.

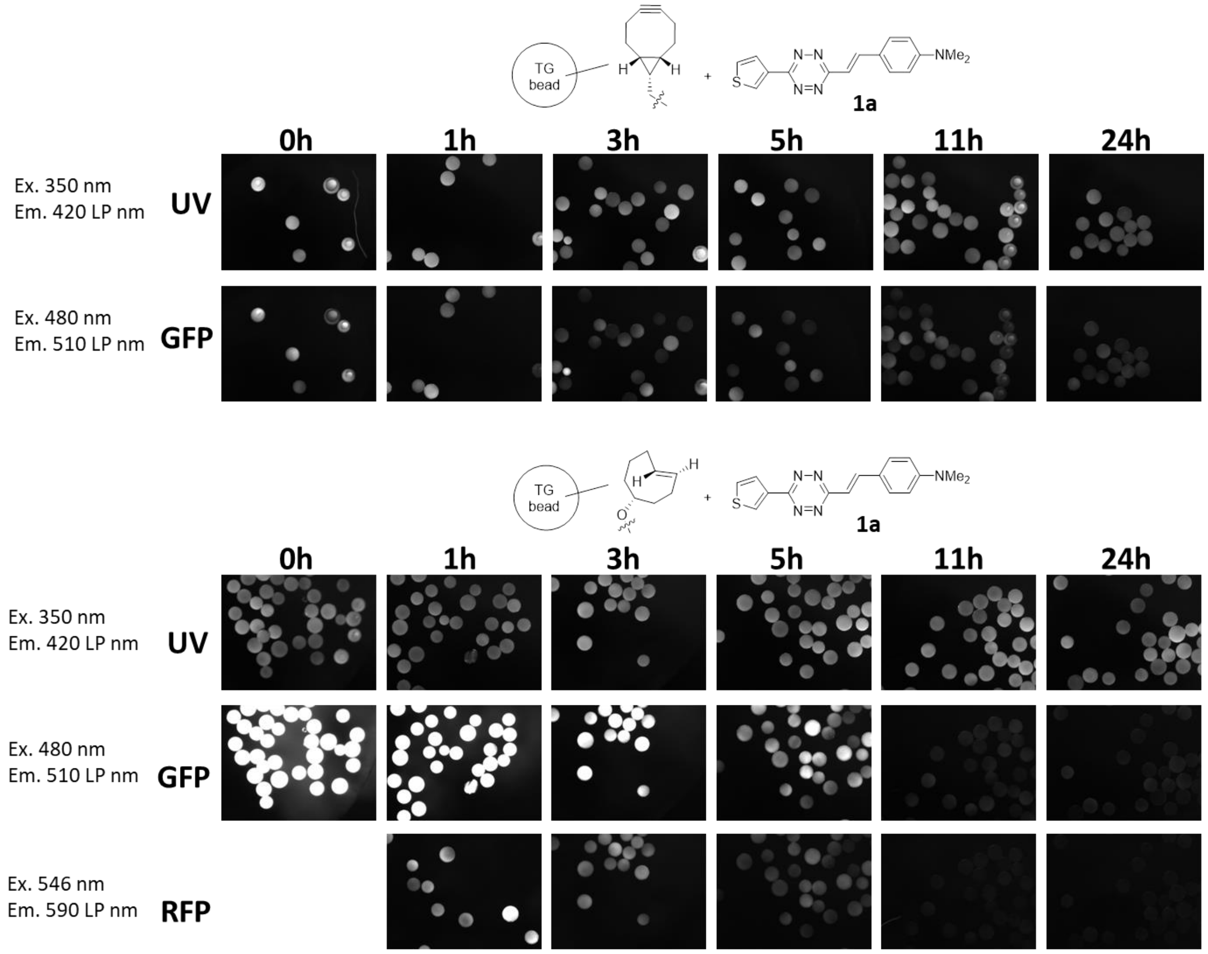

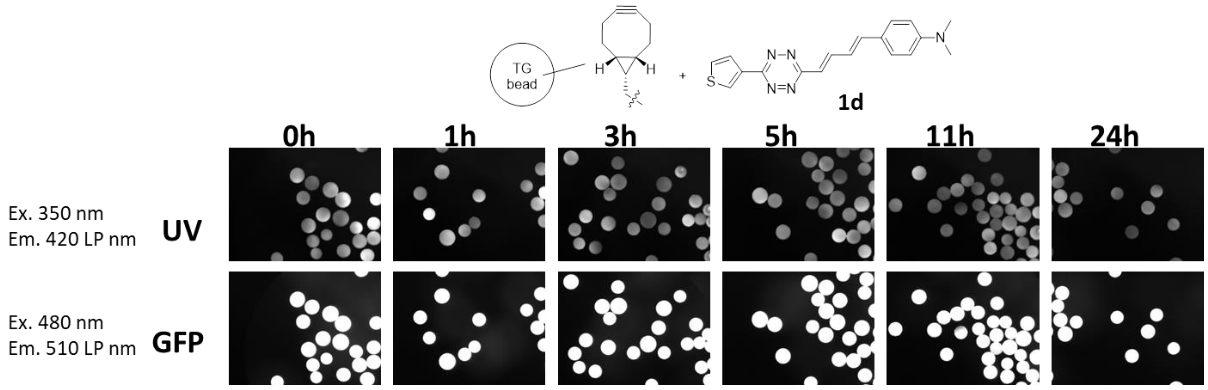
TG bead
1d
0h
1h
3h
5h
11h
24h
Ex. 350 nm
Em. 420 LP nm
UV
Ex. 480 nm
Em. 510 LP nm
GFP

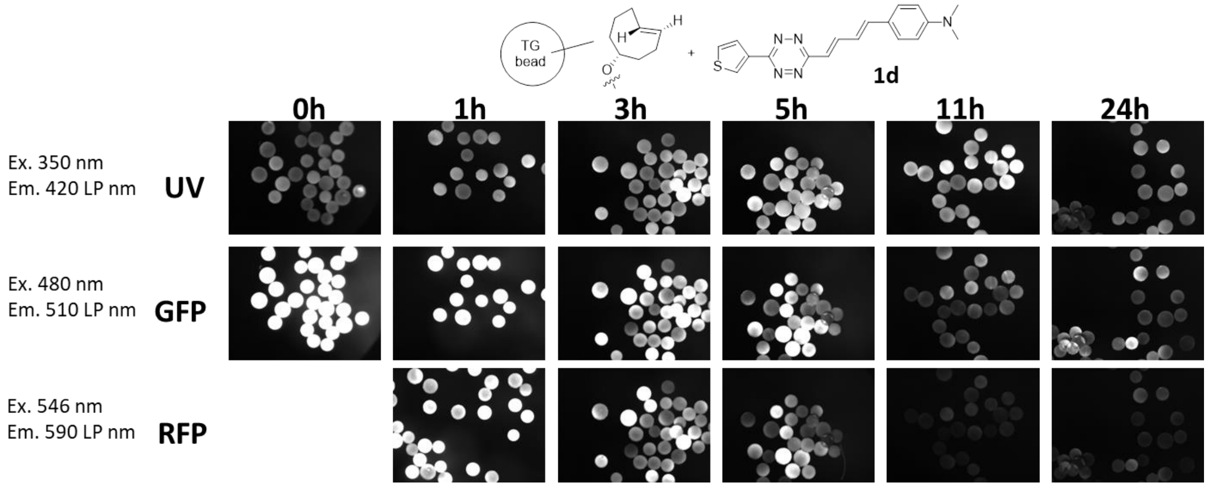
TG bead
1d
0h
1h
3h
5h
11h
24h
Ex. 350 nm
Em. 420 LP nm
UV
Ex. 480 nm
Em. 510 LP nm
GFP
Ex. 546 nm
Em. 590 LP nm
RFP

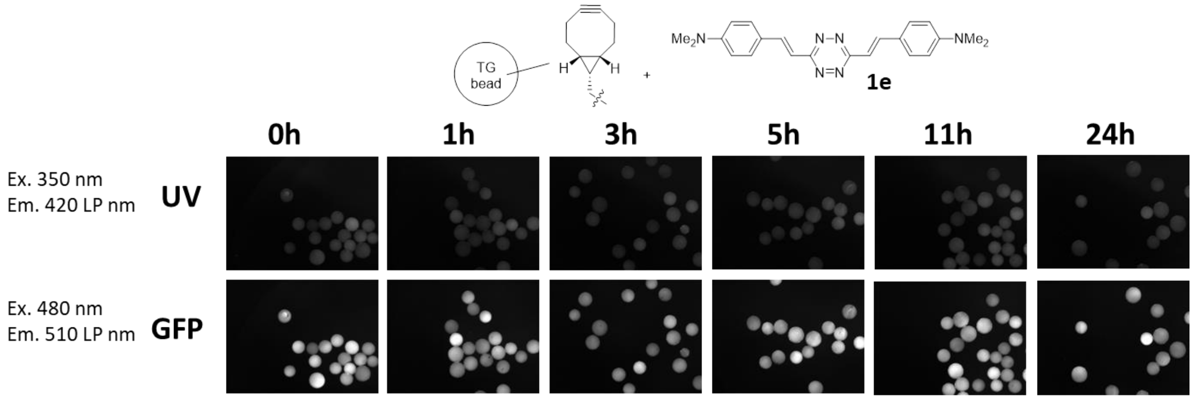
TG bead
1e
0h
1h
3h
5h
11h
24h
Ex. 350 nm
Em. 420 LP nm
UV
Ex. 480 nm
Em. 510 LP nm
GFP

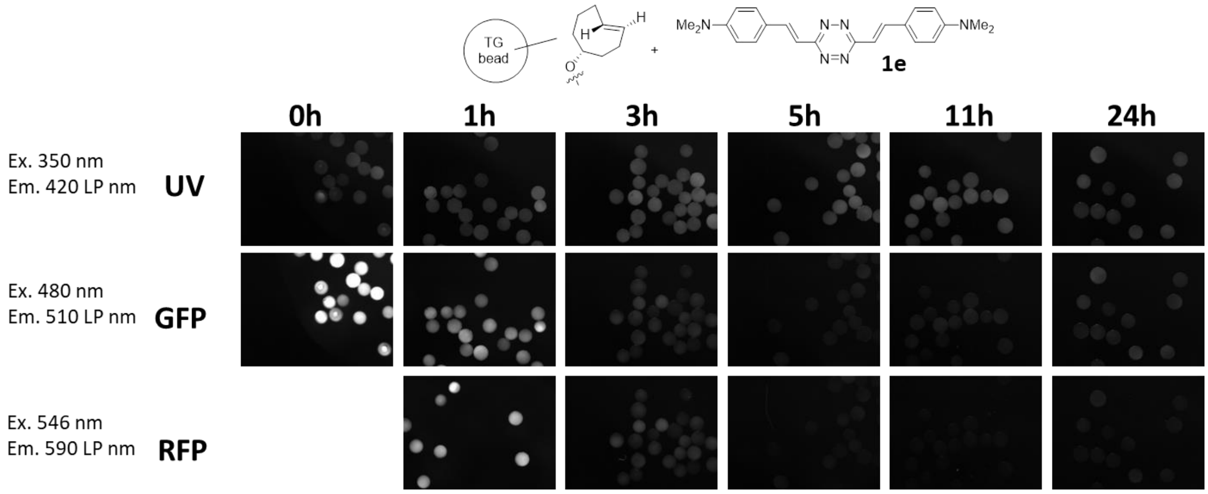
TG bead
1e
0h
1h
3h
5h
11h
24h
Ex. 350 nm
Em. 420 LP nm
UV
Ex. 480 nm
Em. 510 LP nm
GFP
Ex. 546 nm
Em. 590 LP nm
RFP

References

[1] A. Vazquez, R. Dzijak, M. Dracinsky, R. Rampmaier, S. J. Siegl, M. Vrabel, *Angew. Chem. Int. Ed.* **2017**, *56*, 1334-1337.

[2] S. J. Siegl, J. Galeta, R. Dzijak, A. Vazquez, M. Del Río-Villanueva, M. Dracinsky, M. Vrabel, *ChemBioChem*.

[3] M. Beshai, B. Dhudshia, R. Mills, A. N. Thadani, *Tetrahedron Lett* **2008**, *49*, 6794-6796.

[4] H. X. Wu, J. Yang, J. Seckute, N. K. Devaraj, *Angew. Chem. Int. Ed.* **2014**, *53*, 5805-5809.

[5] D. F. Eaton, *J Photochem Photobiol B* **1988**, *2*, 523-531.

[6] J. E. Bertie, Z. D. Lan, *J Phys Chem B* **1997**, *101*, 4111-4119.

[7] F. Bedard, A. Girard, E. Biron, *Int. J. Pept. Res. Ther.* **2013**, *19*, 13-23.

Copies of NMR spectra

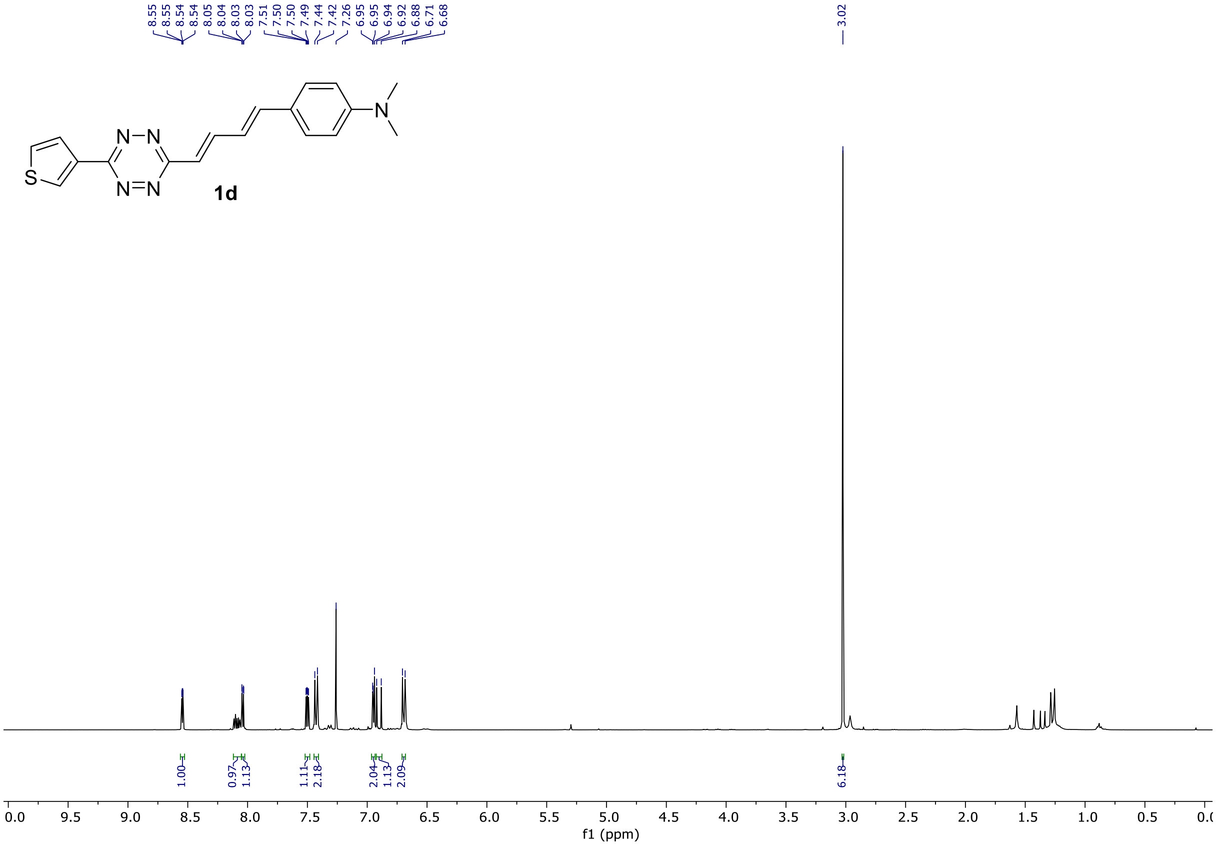

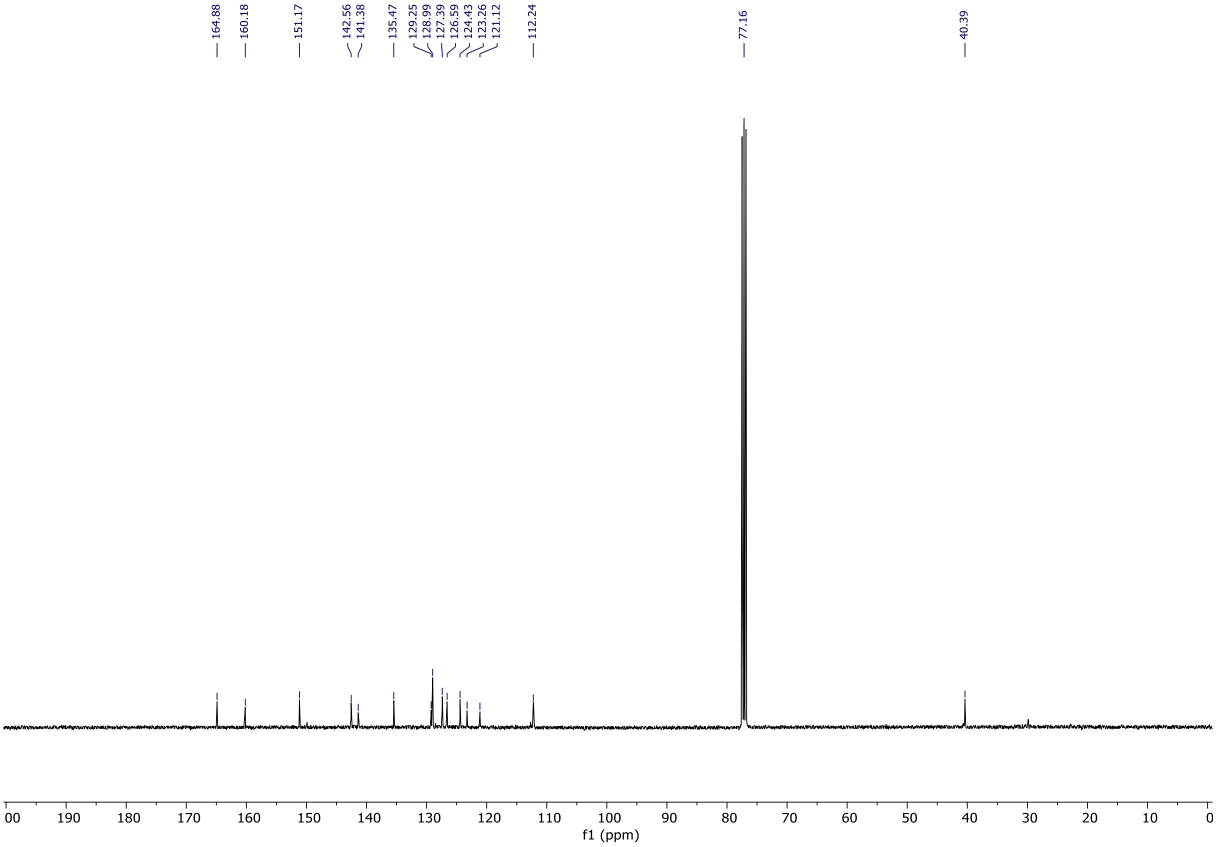

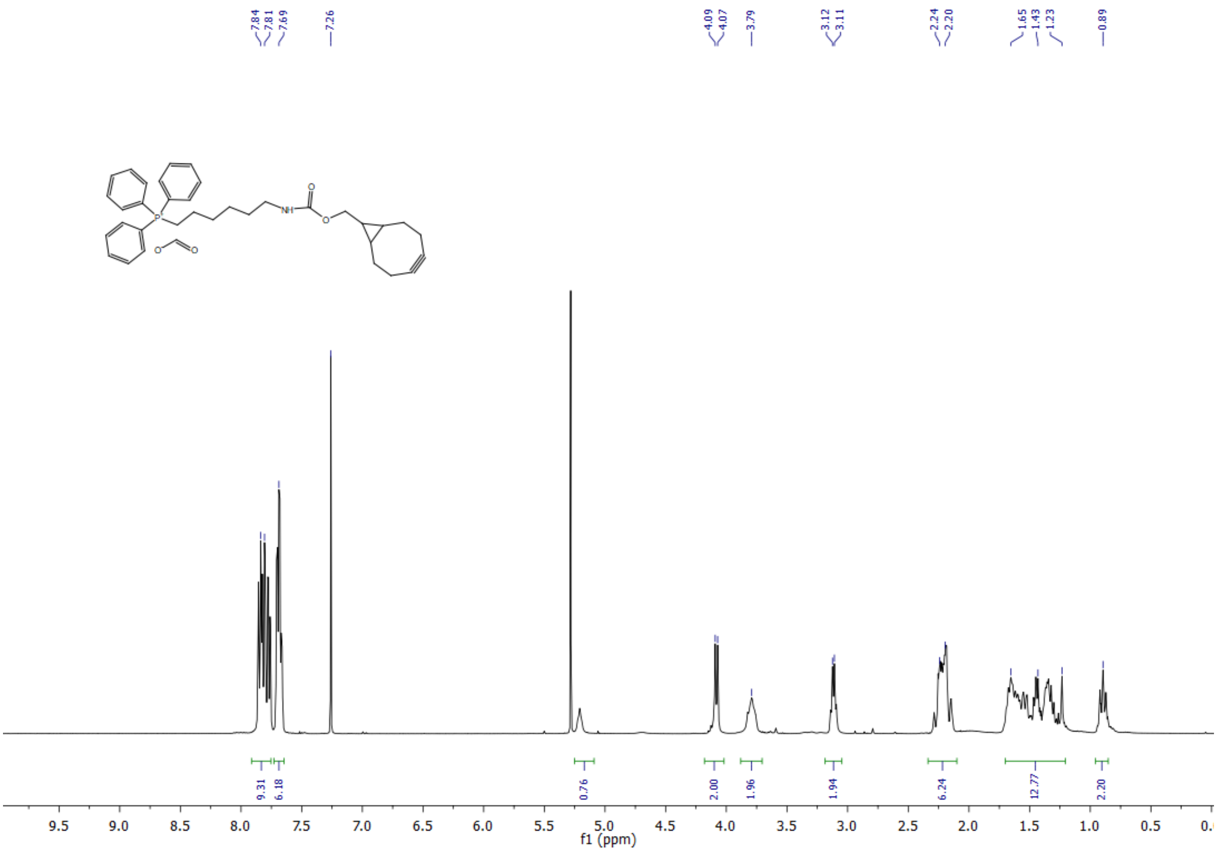

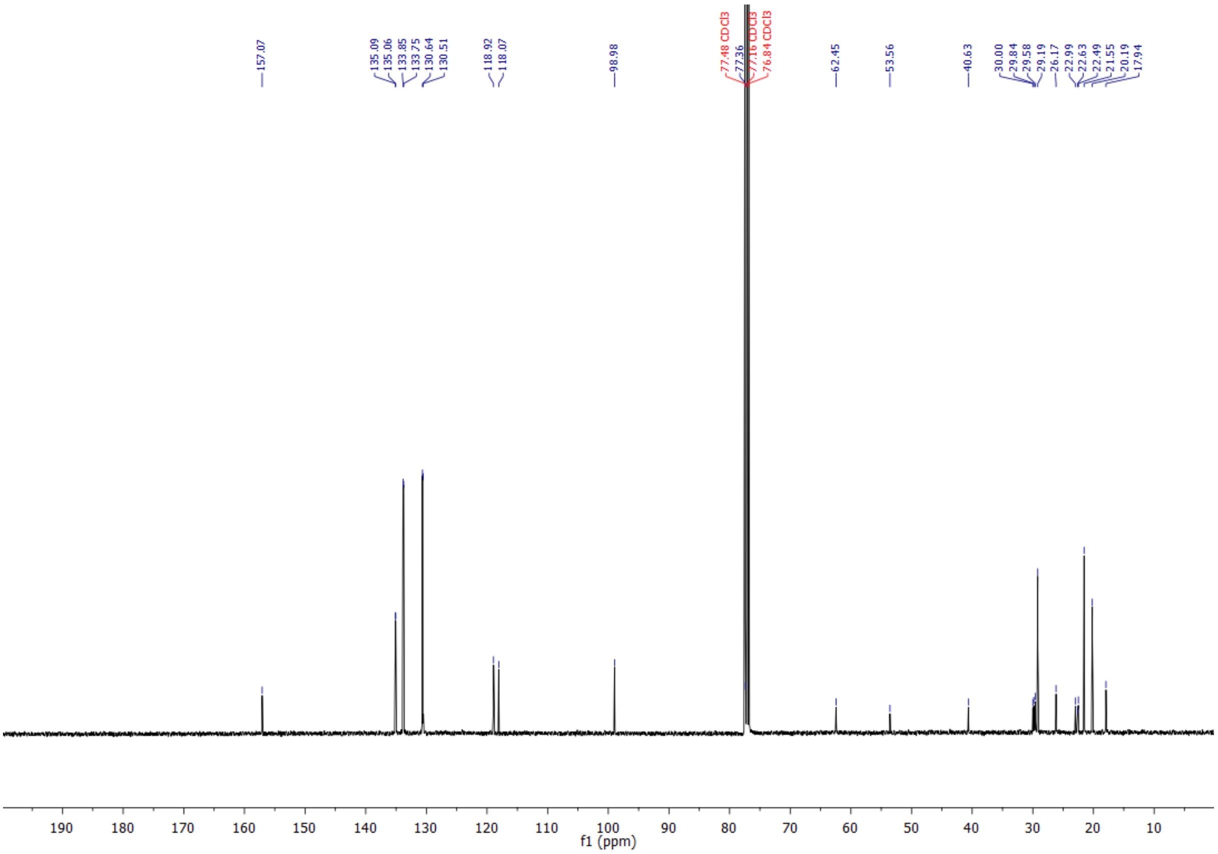

Supplement: Supplementary file 1 — Supplementary [file CPLU-84-493-s001.pdf]
